# Significant effects of host dietary guild and phylogeny in wild lemur gut microbiomes

**DOI:** 10.1038/s43705-022-00115-6

**Published:** 2022-04-08

**Authors:** Mariah E. Donohue, Amanda K. Rowe, Eric Kowalewski, Zoe L. Hert, Carly E. Karrick, Lovasoa J. Randriamanandaza, Francois Zakamanana, Stela Nomenjanahary, Rostant Y. Andriamalala, Kathryn M. Everson, Audrey D. Law, Luke Moe, Patricia C. Wright, David W. Weisrock

**Affiliations:** 1 Department of Biology, University of Kentucky, Lexington, KY USA; 2 Interdepartmental Doctoral Program in Anthropological Sciences, Stony Brook University, Stony Brook, New York, NY USA; 3 Centre ValBio Research Station, Ranomafana, Madagascar; 4 Anthropobiologie et Développement Durable, Université Antananarivo, Antananarivo, Madagascar; 5 Department of Plant and Soil Sciences, University of Kentucky, Lexington, KY USA; 6 Department of Anthropology, Stony Brook University, Stony Brook, New York, NY USA

**Keywords:** Microbiome, Microbial ecology

## Abstract

Mammals harbor diverse gut microbiomes (GMs) that perform critical functions for host health and fitness. Identifying factors associated with GM variation can help illuminate the role of microbial symbionts in mediating host ecological interactions and evolutionary processes, including diversification and adaptation. Many mammals demonstrate phylosymbiosis—a pattern in which more closely-related species harbor more similar GMs—while others show overwhelming influences of diet and habitat. Here, we generated 16S rRNA sequence data from fecal samples of 15 species of wild lemurs across southern Madagascar to (1) test a hypothesis of phylosymbiosis, and (2) test trait correlations between dietary guild, habitat, and GM diversity. Our results provide strong evidence of phylosymbiosis, though some closely-related species with substantial ecological niche overlap exhibited greater GM similarity than expected under Brownian motion. Phylogenetic regressions also showed a significant correlation between dietary guild and UniFrac diversity, but not Bray-Curtis or Jaccard. This discrepancy between beta diversity metrics suggests that older microbial clades have stronger associations with diet than younger clades, as UniFrac weights older clades more heavily. We conclude that GM diversity is predominantly shaped by host phylogeny, and that microbes associated with diet were likely acquired before evolutionary radiations within the lemur families examined.

## Introduction

The mammalian gastrointestinal tract harbors trillions of resident microbes, collectively known as the gut microbiome (GM). The relationship between hosts and their gut microbes is ancient and obligatory, as the GM has evolved to perform critical functions for host immunity and digestion [1]. The initial transmission of gut microbes can be vertical (from parent to offspring) or horizontal (through social or environmental interactions) [2]. Microbes then undergo ecological filtering, where a combination of genetic and environmental factors determine which species colonize and proliferate within the host [3]. Beyond host-mediated selection, GMs are also subject to basic community assembly processes such as niche competition, dispersal, drift, and diversification [3, 4]. Thus, host-GM interactions are shaped by complex ecological and evolutionary factors, although the relative importance of each remains unclear [5].

Comparative analyses of the host-mediated factors influencing GM patterning (i.e., microbial diversity and composition) across host species have produced two primary hypotheses. The phylosymbiosis hypothesis predicts a correlation between host phylogenetic and GM distance, with more closely-related species harboring more similar GMs, irrespective of ecological factors [6]. As an alternative or complementary hypothesis, host ecology—in particular, diet—is thought to play a strong role in structuring GM patterns, with shared ecological adaptations leading to GM similarity across distantly-related species [e.g., 7, 8]. Both hypotheses have garnered support across a range of animal systems, though results within the same clade are sometimes conflicting [9–11]. This is particularly true within primates, where some studies have shown diet to be a more important factor relative to evolutionary history [e.g., 12–17], while the opposite pattern has been found in others [e.g., 18–22].

Our study aims to help resolve the relative importance of host evolutionary history and ecology in lemurs, a sub-order of primates endemic to the island of Madagascar. The lemur ancestor rafted from continental Africa to Madagascar 50–70 million years ago [23–25], where they experienced an adaptive radiation and diversified into over 100 species [25, 26]. Evidence suggests lemurs evolved in an ancestral climate niche characterized by high rainfall and mild seasonality [27]. As time progressed, lemurs dispersed across the island and now occupy nearly every natural habitat, from the lush rainforests of the east to the arid spiny deserts of the west. Fine-scale niche partitioning and remarkable diversity in body size, activity patterns, social systems, and diet allows for high levels of coexistence, with as many as 14 species living in sympatry in some habitats [26]. Simultaneously, closely-related species (e.g., congenerics) can be found in a range of ecosystems with different abiotic conditions and food resources [28].

GM divergence and adaptation may contribute to lemur diversification; however, more research about the interplay between evolution, ecology, and the GM is sorely needed in this system. Previous studies identified significant links between GM composition and lemur ecological factors, including dietary strategy [19, 21, 22, 29–31], habitat [30, 32–34], and season [35, 36]. Lemurs have also been shown to harbor species-specific GMs [14, 21, 22, 30, 35], though the effect of host phylogeny remains ambiguous. For example, one study using a taxonomically diverse lemur dataset found clear signatures of phylosymbiosis [22]. However, a study using intrageneric comparisons within *Eulemur* and *Propithecus* found that effects of local ecology overwhelmed phylogeny [30]. Superior effects of lemur ecology over evolution were also reported in datasets comprised of closely-related allopatric [33] and distantly-related sympatric [14] lemur species. Nonetheless, in a comparative study using captive nocturnal strepsirrhines (a sub-order of primates), including lemurs, strong effects of both dietary strategy and phylogeny were detected [21].

To gain new insight into the factors driving GM patterning in lemurs, we compared diverse lemur communities in Madagascar’s eastern rainforests and western dry forests. These communities provide a natural experiment to test emerging hypotheses about GM patterning in lemurs, as they include sympatric and allopatric species with varying degrees of ecological niche divergence and evolutionary relatedness. We used 16S rRNA sequencing to: (1) test the hypothesis of phylosymbiosis, and (2) test for correlations between the GM and particular ecological traits (dietary guild and habitat). We analyzed our dataset using multiple statistical approaches to determine whether interpretations of GM patterning changed, especially in comparisons between different beta diversity metrics and phylogenetically-informed and uninformed methods.

## Methods

### Ethics statement

Methods for fecal collections of wild primates were approved by the IACUC committees of the University of Kentucky (IACUC #2018-2919) and Stony Brook University (IACUC #1177457-3 and #11323621-2). Field protocols were approved by Madagascar National Parks and by the Ministere de l’Environment et des Eaux et Forets.

### Study sites and sample collection

**Fig. 1:**
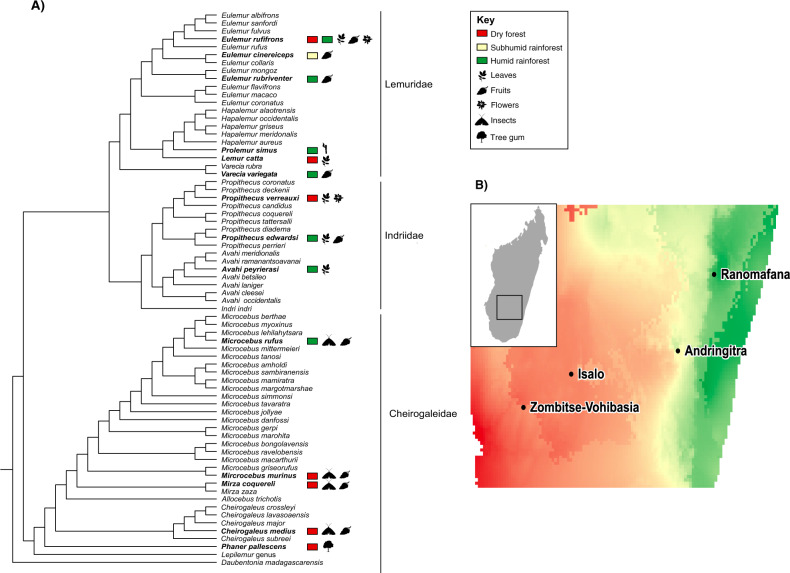
The phylogenetic and ecological diversity of lemur species sampled in this study. **A** Lemur phylogeny adapted from Herrera and Dávalos [25]. Bolded tip labels denote species sampled in our study. Colors within squares represent the habitat of the collection site (see Fig. 1B); icons signify food groups that account for at least 20% of the species’ diet (see Table S1). **B** Map of sampling sites, colored by values provided in the WorldClim “Precipitation of Warmest Quarter” raster layer [59].

We collected fecal samples from 15 wild lemur species representing ten genera and three families (Fig. 1A; Table 1) occupying diverse habitats across southern Madagascar (Fig. 1B). For large-bodied lemur species, we collected fecal samples within one minute of deposition during non-invasive behavioral follows. Small-bodied lemur species were live-captured and fecal samples were collected from inside the trap or from the animal [37]. Samples were collected using sterilized tweezers, placed in tubes with 96% ethanol, and stored at room temperature pending laboratory processing.

**Table 1:** Taxonomic identification, site, and sampling of study subjects. Only includes samples that were retained after bioinformatic filtering.

Host family	Host species	*n*		Site	Sampling dates
		Individuals	Samples		
Cheirogaleidae	*Cheirogaleus medius*	11	11	Zombitse	12/30/18–1/16/19
	*Microcebus murinus*	1	1	Zombitse	12/30/18–1/16/19
	*Microcebus rufus*	30	30	Ranomafana	6/1/18–7/21/18
	*Mirza coquereli*	9	9	Zombitse	12/30/18–1/16/19
	*Phaner pallescens*	6	6	Zombitse	12/30/18–1/16/19
Lemuridae	*Eulemur rubriventer*	14	16	Ranomafana	6/1/18–7/21/18
	*Eulemur rufifrons* (east)	18	18	Ranomafana	6/1/18–7/21/18
	*Eulemur rufifrons* (west)	10	12	Isalo	7/5/18–7/10/18
	*E. rufifrons x E. cinereiceps* hybrids	7	7	Andringitra	5/20/19–6/30/19
	*Lemur catta*	17	17	Isalo	7/5/18–7/10/18
	*Prolemur simus*	2	3	Ranomafana	7/1/19–7/20/19
	*Varecia variegata*	4	4	Ranomafana	7/1/19–7/20/19
Indriidae	*Avahi peyrierasi*	6	6	Ranomafana	6/1/18–7/21/18
	*Propithecus edwardsi*	7	13	Ranomafana	6/1/18–7/21/18
	*Propithecus verreauxi*	6	7	Isalo	7/5/18–7/10/18

Samples were acquired during four expeditions between May 2018 and July 2019 (Table 1). To maximize ecological variation, we selected eastern rainforest sites (Andringitra National Park: 700–2658 m; Ranomafana National Park: elevation 500–1500 m), and western dry forest sites (Isalo National Park: elevation 510–1268 m; Zombitse-Vohibasia National Park: elevation 300–825 m; Fig. 1B).

### DNA sequence generation

We generated microbial DNA sequence data from 172 fecal samples. DNA was extracted from ~0.2 g of feces per sample using the Qiagen QIAamp^®^ PowerFecal^®^ Kit (Hilden, Germany) following the manufacturer protocol. The V4 region of the 16S rRNA gene was PCR amplified from each sample in duplicate using dual-indexed primers [38]. PCRs contained 10 µL of Extract-N-Amp™ PCR reaction mix (Sigma-Aldrich; St. Louis, MO, USA), 0.5 µL each of forward and reverse primers, and 4 µL of DNA template. PCR negative controls (PCR reactions without fecal DNA template) were performed for all PCR batches using unique dual-indexed primers. PCR amplification was performed in a thermal cycler with a 3 min initial denaturation at 95 °C followed by 34 cycles of 30 s at 95 °C, 1 min at 50°C, and 1 min at 72 °C, and a final extension of 2 min at 72 °C. To control for batch effects, DNA extractions and PCRs were randomized across species and sites.

Size and quality of PCR products were confirmed using agarose gel electrophoresis. Libraries were normalized using the SequalPrep™ Normalization Plate Kit (ThermoFisher; Waltham, MA, USA) and quantified with a Qubit^®^ fluorometer. After confirming each sample had a DNA concentration of ~1 ng/µL, samples were pooled and submitted to the UK HealthCare Genomics Core for sequencing on an Illumina MiSeq flowcell using a v2 reagent kit and 250 bp paired-end reads. Sequences were demultiplexed using the Illumina MiSeq pipeline.

### Bioinformatics

Illumina sequencing reads were filtered and processed using the DADA2 pipeline [39] in QIIME2 v. 2018.11 [40]. The 16S rRNA amplicon sequence variants (hereafter ASVs) were identified using the SILVA reference database version 132 [41]. ASVs were clustered using a 99% similarity threshold. As is common in microbiome research, our PCR negative controls were contaminated with microbial DNA [42]. To reduce the potential impacts of this contamination, we filtered all ASVs found in PCR negative controls from our lemur samples (Table S1).

To determine the minimum number of reads required to accurately estimate GM diversity, we generated a rarefaction curve in QIIME2 (Fig. S1). PCR negative controls (*n* = 10) and samples with less than 2000 reads (*n* = 4) were removed from downstream analyses, leaving 158 samples total.

### Characterization of GM diversity

Diversity analyses were completed in QIIME2 with rarefaction at 2000 sequences per sample. We calculated GM diversity using an ASV table (a type of QIIME2 file) with sequence data for all 158 remaining samples. Our analyses included four standard beta diversity metrics, all of which quantify differences in microbial species composition between samples: unweighted UniFrac, weighted UniFrac, Bray-Curtis, and Jaccard. UniFrac metrics are phylogenetic measures of diversity which, by design, consider phylogenetic relatedness and branch length in comparisons between communities [43]. This effectively minimizes the influence of recent microbial evolution in community divergence, as clades with longer branch lengths (i.e., deeper evolutionary history) are weighted. Bray-Curtis and Jaccard, on the other hand, use microbial taxonomy (not phylogeny) to measure community dissimilarity and similarity, respectively. As a result, branch lengths and relatedness between species are not considered, making these metrics more sensitive to recent microbial evolution as all species are weighted equally [44]. Further, while Bray-Curtis and weighted UniFrac incorporate abundance of ASVs (i.e., weighted beta diversity metrics), unweighted UniFrac and Jaccard only consider presence/absence (i.e., unweighted beta diversity metrics). All four metrics have known strengths and weaknesses for phylosymbiosis analyses, and leveraging these differences can help identify drivers of community divergence—especially the importance of ancient (more significant using UniFrac) versus nascent (more significant using star phylogeny) microbial clades, and presence/absence (more significant using unweighted metrics) versus relative abundance (more significant using weighted metrics) of specific microbial species [44].

Beta diversity was visualized using PCoA plots created with phyloseq [45]. We chose PCoA—a metric multidimensional scaling method—over non-metric (NMDS) because results are fixed, even after re-analysis. Additionally, PCoA does not scale down the distribution of samples in ordination space, allowing for a greater number of axes [46].

We also included a measure of alpha diversity, which quantifies the number of microbial species in a given sample. For this analysis, we used Faith’s phylogenetic diversity (PD), as it accounts for the evolutionary relatedness of communities and allows us to ask whether closely-related lemur species host GMs with similar phylogenetic diversity. Alpha diversity was visualized with a boxplot created using ggplot2 [47] (Fig. S2).

### Testing phylosymbiosis

The phylosymbiosis hypothesis makes a number of predictions that can be tested using trees depicting the similarity of the GM and the phylogeny of the host species. Therefore, the number of samples in the GM dataset must match the number of tips in the host phylogeny. To reduce the GM dataset such that one GM community represents each lemur species, we generated unweighted pair group method with arithmetic mean (UPGMA) dendrograms for each beta diversity metric by both (1) randomly picking one sample per species and (2) calculating the average frequency of each ASV across samples within a given species using the “mean ceiling” method. Random picking was repeated 10 times per species without replacement, unless a species had fewer than 10 samples, in which case some samples were used in multiple trials. For the host species phylogeny, we used a Bayesian fossilized birth death tree from [25]. This tree was downloaded from Dryad (10.5061/dryad.51f00) and pruned to include only the taxa in this study using the “drop.tip” function in the R package ape [48].

We inferred the phylogenetic placement of lemur taxa not included in the host phylogeny [25] by renaming the tips of their closest available relatives. Specifically, we coded *Phaner pallescens* as *Phaner furcifer* and *E. rufifrons* x *E. cinereiceps* hybrids as *E. cinereiceps*. Because we collected fecal samples from two allopatric populations of *E. rufifrons* (one from the eastern rainforest and one from the western dry forest), we coded the western population as *E. fulvus*. These inferences are imperfect, as true divergence times and genetic distances, particularly within *Eulemur*, are almost certainly smaller. However, phylogenetic data for these populations are not currently available. The pruned phylogeny with edited tip labels was used for all subsequent tests of phylosymbiosis.

One prediction of phylosymbiosis is that branching patterns of GM community similarity recapitulate host branching order. To test this, we used a “co-dendrogram” approach to visualize congruence between host phylogeny and GM dendrograms using the phytools “cophylo” command with rotation [49]. Topological congruence was quantified using normalized Robinson-Foulds (RF) distance [50] with a range from 0 (complete congruence) to 1 (complete incongruence). RF distances were calculated in R using the “RF.dist” function in phangorn [51]. Significance was calculated by comparing the observed RF distance to RF distances from 10,000 randomized GM trees [6] using the “rtree” function in ape.

A second phylosymbiosis prediction is that as phylogenetic distance between hosts increases, so too does GM distance. To test this prediction, we performed Mantel tests using a patristic distance matrix (i.e., sum of branch lengths), computed using the “distTips” function in the R package adephylo [52] based on the host phylogeny and beta diversity distance matrices derived from UPGMA trees. Statistical significance was calculated with the “mantel” function in the R package vegan [53] using the Pearson method and 999 permutations.

Finally, though tested more rarely, phylosymbiosis predicts that the GM exhibits phylogenetic signal [54]. Currently available tests of phylogenetic signal require reducing the dimensionality of GM beta diversity to a single univariate value for each host species. To do this, we performed principal coordinate analyses (PCoAs) for each beta diversity measure (weighted UniFrac, unweighted uniFrac, Bray-Curtis, and Jaccard). We then used two methods to test for phylogenetic signal on each of the first five principal coordinates (PCos), in addition to alpha diversity, as represented using Faith’s PD. First, we calculated Blomberg’s *K* [55], a variance ratio that determines if closely-related species resemble each other more or less than expected under Brownian motion models of evolution (random drift); *K* values greater than 1 indicate greater similarity while values less than 1 indicate greater dissimilarity. Second, we estimated Pagel’s Lambda [56], a scaling parameter that also assumes Brownian evolution, with values ranging from 0 (trait evolved independent of phylogeny) to 1 (trait evolution corresponds to Brownian motion). Both measures were assessed using the “phylosig” function in the R package geiger [57].

### Dietary and habitat data

We gleaned dietary guild (hereafter “diet”) data from previous studies that estimated the percent of total feeding time each species was observed consuming one of six major food items (fruit, leaves, insects, bamboo, gum, flowers; Table S2). When possible, we preferentially selected studies that were conducted at the same locality as our fecal collections.

We built ecological niche models (ENMs) to represent the preferred habitat for each lemur species. Briefly, ENMs combine occurrence data with environmental data to build correlative models of habitat suitability and ecological requirements [58].

ENMs were constructed in Maxent v3.4.1 [58] with environmental layers downloaded from the WorldClim database [59; see Supporting Information] and presence localities acquired from this study and GBIF.org. Differences in habitat suitability were assessed using the predicted environmental values for each species, averaged across replicates. These predicted environmental values were input in downstream trait correlation models (see below).

### Testing ecological correlates of GM diversity

We tested trait correlations between GM diversity and ecological traits—specifically diet, habitat, and the interaction of diet and habitat. We performed PCAs on diet (proportion of time devoted to each food) and habitat data (environmental values output from ENMs) to extract the eigenvalues associated with PC1. These eigenvalues were then input as predictor variables in phylogenetic generalized least squares (PGLS) and Adonis regression models, using the R package caper [60] and QIIME2, respectively. We ran five models per regression method, each using a different GM diversity metric (Bray-Curtis, Jaccard, unweighted UniFrac, weighted UniFrac, or Faith’s PD) as the response variable. We used Adonis regressions to tease apart the relative effects of diet, habitat, and host taxonomy, using the formula “Diet_PC1*Habitat_PC1 + Family/Genus/Species” as the independent variable. We used PGLS regressions to determine whether ecological traits of interest (diet and habitat and their interaction) were significant after accounting for the host phylogeny. PGLS models are univariate; therefore, beta diversity metrics were represented using the eigenvalues associated with the first PCo. Adonis tests can accept multivariate response variables in QIIME2, so beta diversity did not need to be reduced. Adonis tests were run with 999 permutations using additive sums of squares and checked for homogeneity of variance.

To determine whether these traits exhibit phylogenetic structure, we also measured Blomberg’s K and Pagel’s lambda for diet and habitat.

## Results

We generated a total of 10,198,053 sequence reads (mean: 61,066 per sample; range: 280 to 268,331). After filtering, we retained 9,872,906 sequence reads, with a mean of 62,094 (range: 3270 to 268,331) per sample. Averages taken across samples showed Bacteroidetes was generally the most abundant phylum in our dataset (35%), followed by Firmicutes (25%), Proteobacteria (13%), Actinobacteria (7%), Spirochetes (6%), Cyanobacteria (5%), and Verrucomicrobia (3%). Low-abundance phyla (<2%) accounted for about 6% of phylum-level microbial diversity (Fig. S3).

### Tests of phylosymbiosis

**Fig. 2:**
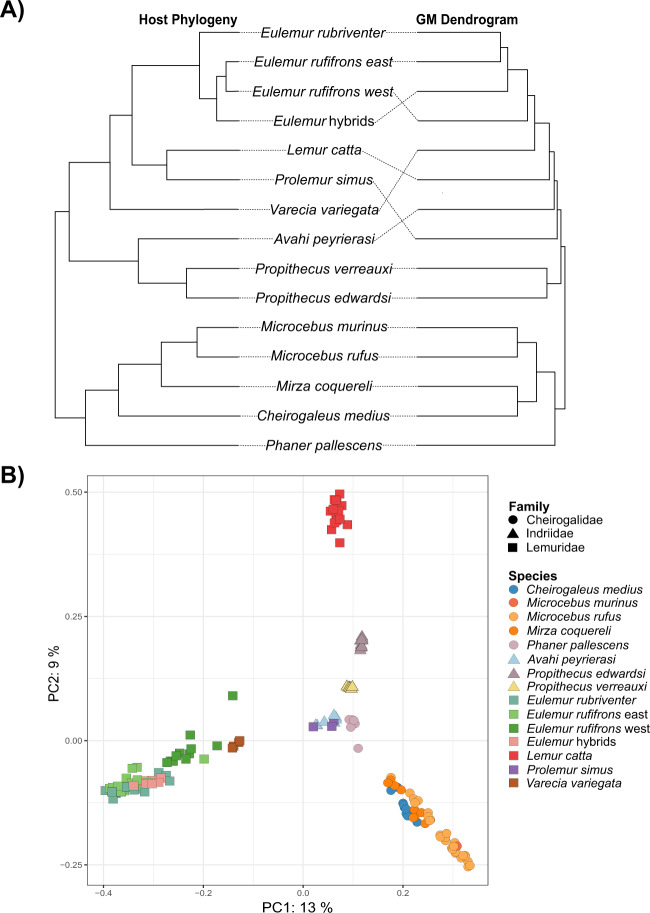
Host phylogenetic relatedness and taxonomic identity significantly contribute to differences in GM beta diversity across samples. **A** Co-dendrogram comparison of branching order between the host phylogeny (left) and species-average Jaccard UPGMA tree. **B** Jaccard PCoA plot coded by host family (shape) and species (color).

We consistently found evidence in support of phylosymbiosis using co-dendrogram and correlation-based analyses, regardless of the method employed for selecting representative species GMs (i.e., randomly selected or species-average GM communities) or diversity metric. RF distances between GM dendrograms and the lemur phylogeny ranged between 0.50 and 0.92 (average = 0.67 ± 0.12), with all co-dendrograms showing significantly higher congruence than expected by chance (Figs. 2A; S4; Tables 2; S3). We also found a positive correlation between host phylogenetic distance and GM distance, with R-values ranging from 0.17 to 0.76 (average = 0.53 ± 0.15) and 42 of 44 total Mantel tests being statistically significant (Tables 2; S4).

**Table 2:** Results of co-dendrogram and Mantel tests of phylosymbiosis using species-average GM data.

Distance matrix	Co-dendrogram		Correlation	
	RF	*p*	Mantel r	*p*
Bray-Curtis	0.67	<0.0001*	0.49	0.003*
Jaccard	0.58	<0.0001*	0.67	0.001*
Unweighted UniFrac	0.75	<0.0001*	0.68	0.001*
Weighted UniFrac	0.75	<0.0001*	0.56	0.001*

*p value < 0.05.

**Fig. 3:**
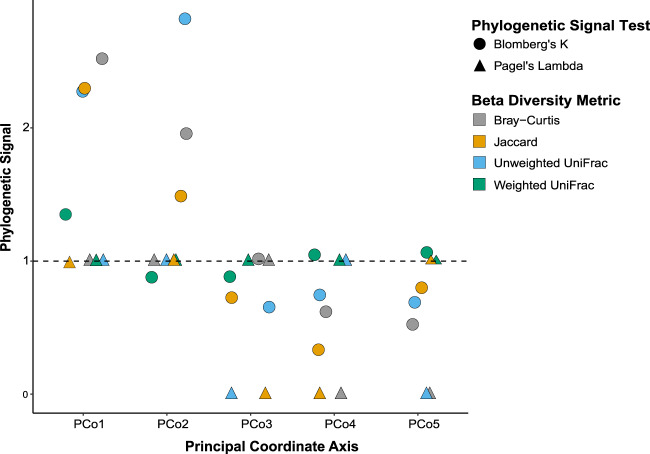
Lemur GM beta diversity shows significant phylogenetic signal. Here, we demonstrate strength of phylogenetic signal (*y*-axis) across species-average principal coordinate scores (*x*-axis). We compared beta diversity metrics (colors) and phylogenetic signal tests (shapes). The horizontal dashed line marks the maximum possible Pagel’s Lambda score (strong phylogenetic signal), as well as the minimum threshold to conclude significant phylogenetic signal using Blomberg’s *K*.

Lending further support for phylosymbiosis, we also detected significant phylogenetic signal (Fig. 3, Table S5). PCo1 was significant in the host species-average dataset and every replicate using Bray-Curtis, Jaccard, and unweighted UniFrac. Using weighted UniFrac, PCo1 was significant in the species-average dataset, and five Blomberg’s *K* and Pagel’s lambda replicates each. This trend continued across all PCo axes, with generally higher phylogenetic signal and congruence in Blomberg’s *K* values for unweighted UniFrac, Bray-Curtis, and Jaccard than weighted UniFrac. However, using Pagel’s lambda, unweighted UniFrac values were lower than those for weighted UniFrac, Jaccard, and Bray-Curtis. Faith’s PD also exhibited significant phylogenetic signal, though randomly selected communities had a weaker signal than the species-averaged dataset.

### Dietary and habitat variation

Dietary guild differed across lemur species (Table S2). Fruit was included in the diet of most species sampled (13 of 15). Five species were shown to regularly consume insects. Seven species regularly consumed leaves. Gummivory was observed in just two species (*Microcebus murinus* and *Phaner pallescens*), both of which also consume fruits and insects. One species, *Prolemur simus*, specialized on cyanide-rich bamboo. PCA of dietary data resulted in a PC1 that explained 35.42% of the variance. PC1 was primarily explained by proportion of insects and leaves (Fig. S5).

ENMs showed that habitat suitability differed across species and reflected known distributions. (Fig. S6). PCA of the ENM data resulted in a PC1 that explained 80.51% of the variance. The two most significant variables driving PC1 were maximum temperature of the warmest month and precipitation of the warmest quarter (Fig. S7).

### Tests of associations with the GM

PGLS regressions showed that diet was a significant predictor of GM beta diversity using UniFrac metrics, but not Jaccard or Bray-Curtis (Tables 3; S8). More specifically, diet was significantly correlated with unweighted and weighted UniFrac beta diversity in seven and four of 10 randomly selected communities, respectively, in addition to the host species-average datasets. Diet was not significant using the species-averaged Bray-Curtis or Jaccard datasets, and was only significant in one Bray-Curtis replicate. Habitat was not a significant predictor of GM beta diversity, with the exception of three randomly selected UniFrac datasets. The interaction of diet and habitat was never a significant predictor of beta diversity.

**Table 3:** Comparing the strength of trait correlations across beta diversity metrics using species-average GM data. For these analyses, only the eigenvalues associated with the first principal components (i.e., PC1) for GM beta diversity, diet, and habitat were considered.

Metric	Predictor p-value			Model p-value	Model AIC value
	Diet	Habitat	Diet*Habitat		
Bray-Curtis	0.364	0.611	0.721	0.52	-9.17
Jaccard	0.503	0.316	0.84	0.37	-11.97
Unweighted UniFrac	0.021*	0.419	0.574	0.09	-21.05
Weighted UniFrac	0.012*	0.488	0.747	0.01*	-33.69

^*^p value < 0.05.

Diet was also a significant predictor of Faith’s PD in the host species-average dataset and six random replicates (Table S8). Habitat alone and the interaction of diet and habitat were significant in one and two random replicates, respectively.

Adonis results showed significant effects of both diet and habitat on the GM for all four beta diversity metrics. In each analysis, the effects of diet were stronger than habitat. Diet explained a greater percentage of the total variation for UniFrac metrics (unweighted: 13%; weighted 15%) than star phylogeny metrics (Bray-Curtis: 7%; Jaccard: 6%). Host family and genus had the highest explanatory value across metrics (between 16% and 22%), while host species had the lowest explanatory value, never accounting for more than 2% of the variation (Table S6). Tests for homogeneity of variance based on family, genus, and species were significant using unweighted UniFrac metrics; Jaccard was also significant at the genus level. These results may indicate that differences in microbial communities detected using Adonis tests could stem from differences in within-group dispersions, as opposed to variation in centroid position. Homogeneity of variance was not significant for diet or habitat (Table S7).

PCoA plots showed that clusters were most strongly associated with host taxonomic family, while samples became more overlapping at the genus and species levels (Figs. 2B; S8). Habitat did not have any effect on clustering patterns, and diet, though it may explain some clustering, did not have as strong an effect as taxonomy (Fig. S9).

Diet exhibited significant phylogenetic signal using Pagel’s lambda (*λ* = 0.73; *p* = 0.03), and approached significance using Blomberg’s *K* (*K* = 0.69; *p* = 0.08). Neither measure showed evidence of phylogenetic signal for habitat (Blomberg’s *K* = 0.29, *p* = 0.79; *λ* < 0.0001, p = 1).

## Discussion

We provide evidence for a strong effect of phylogeny in the GM composition of wild lemurs. All tests of phylosymbiosis supported this conclusion. While each measure used has its own set of limitations, we interpret their congruence as indicating that qualitative deficiencies or statistical limitations are unlikely to overwhelm the true evolutionary signal in this dataset. Our results also suggest that, as demonstrated in other mammalian taxa [61], microbes associated with diet were likely acquired at deeper evolutionary time scales than those associated with more recent host speciation events. Thus, the lemur GM is largely shaped by phylogenetic and dietary factors, both of which overwhelmed the effects of habitat in this dataset.

### Phylosymbiosis in lemur GM composition

It is becoming increasingly apparent that no single factor can explain GM patterning, leading to debates about the relative importance of ecology and evolution in shaping the mammalian GM [62]. Many comparative studies in mammals have detected phylosymbiosis [e.g., 61, 63–65]. However, many other studies report no or weak evidence of phylosymbiosis [e.g., 7, 30, 66]. Among lemurs, most studies exploring GM composition have done so at the individual, group, or population level. While valuable for identifying the effects of local ecology [e.g., 32, 34, 35] and social behavior [e.g., 67, 68], understanding GM variation in the larger context of evolutionary history requires multi-species datasets.

Our study, leveraging GM sampling across multiple species in a phylogenetic framework, identified clear evidence for phylosymbiosis. Our data comprised both sympatric and allopatric species, with variation in local habitat and dietary composition. Yet, even with these environmental variables, phylogeny was consistently a significant explainer of GM composition.

At least two previous studies have also identified phylosymbiosis in the lemur GM [21, 22]. While differences in study design exist across these studies and ours, the signature of phylosymbiosis in lemurs appears to be robust to technical variation. Other comparative studies, however, have not found a strong link between evolutionary relatedness and the lemur GM [e.g., 14, 30, 33]. This may be explained by differences in lemur taxonomic diversity, sampling breadth, and/or idiosyncratic patterns in particular clades [18]. For example, within our own dataset and [22], very strong and evident clustering patterns in GM composition are seen at the family level, but samples become much more overlapping among genera and species (Figs. 2B; S5). Similarly, patterns of discordance between host and GM trees increase at shallower levels of evolutionary divergence (Fig. 2A; S4). Thus, studies that include species with deep evolutionary divergence (i.e., different taxonomic families) are probably more likely to detect phylosymbiosis than studies that limit sampling to closely-related species (i.e., congenerics).

Our results also show that in some clades, ecological factors may offer a more parsimonious interpretation of GM patterns than evolutionary history, thus weakening the phylosymbiosis signal. This is particularly true within the Lemuridae family. Co-dendrogram analyses showed rainforest *E. rufifrons* had greater GM similarity to *E. rubriventer* and *E. rufifrons* × *E. cinereiceps* hybrids than to their dry forest intraspecifics (Figs. 2A; S4). Interestingly, *E. rubriventer* and rainforest *E. rufifrons* populations are sympatric, consume many of the same food resources, and occupy the same trees [69]. Such intense ecological niche overlap, coupled with relatively close evolutionary relatedness, may drive greater GM similarity than expected under phylosymbiosis. Further, *Eulemur* hybrids and rainforest *E. rufifrons* occupy similar habitats and share a complicated evolutionary history, wherein secondary contact between *E. cinereiceps* and *E. rufifrons* led to the formation of the incipient hybrid species sampled herein [70, 71]. Hybridization resulted in a genetic exchange that may have extended to the GM, further blurring the ecological, evolutionary, and microbial distinctions between these two species; however, more research in this system is needed.

*Lemur catta* and *Prolemur simus* also challenged the phylosymbiosis hypothesis. Despite close phylogenetic relatedness, these species occupy drastically different habitats and have almost no dietary overlap, as *P. simus* has adapted to primarily subsist on highly toxic bamboo. *Lemur catta* is also more terrestrial and has larger group sizes with stricter dominance hierarchies than any other lemurid, all of which may help justify its distinct GM (Fig. 2B). These striking ecological differences may explain why their GMs did not cluster together in ordination space (Fig. 2B) and were never placed in the same clade in UPGMA dendrograms (Figs. 2A; S4).

Deviations from phylosymbiosis were also found within Cheirogaleidae, with most UPGMA dendrograms placing *Mirza coquereli* and *Cheirogaleus medius* GMs sister to one another, while the host phylogeny shows *M. coquereli* shares more recent common ancestry with the *Microcebus* clade (Figs. 2A; S4). Their GM similarity may stem from occupying the same forest strata, competing for similar food resources, and having more similar body sizes than *Microcebus*, which are much smaller [72]. In contrast, within Indriidae, *Propithecus edwardsi* GM composition was more similar to the allopatric and more closely-related *P. verreauxi*, relative to sympatric *Avahi peyriearsi*. A number of factors may drive stronger phylosymbiosis in Indriidae, including deeper divergence times and allopatric distribution (and, consequently, absence of niche competition) in *Propithecus*. In addition, significant physiological and ecological differences exist between sympatric *Propithecus* and *Avahi; Avahi* is small-bodied, nocturnal, and folivorous, while *Propithecus* is large-bodied, diurnal, and consumes a more varied diet of fruits, leaves, and flowers (Table S2). Thus, greater biological differentiation likely amplifies phylogenetic signal in the GM.

Of course, this discussion has been limited to host-mediated evolutionary and ecological explanations of the GM, but many other factors that could not be examined herein, such as microbe-microbe competition, are also at play. We were also not able to control for seasonality, as all but one cheirogalid species (*Microcebus rufus*) was sampled during the warm/rainy season while indriid and lemurid samples were collected during the cold/dry season. Nonetheless, *Microcebus rufus* and *Microcebus murinus* GMs were sister to each other in most UPGMA dendrograms, indicating that phylogenetic effects outweigh seasonal effects in this clade (Figs. 2B; S4). Future studies should aim to compare strength of phylosymbiosis across seasons, as many lemur species are known to experience dramatic dietary shifts during seasonal shortages of preferred foods, such as fruits [28].

### Contributors to phylosymbiosis patterning

It has become well-understood that phylosymbiosis can arise, at least in part, from host-mediated selection. Among the many host-mediated factors that can contribute to phylosymbiosis, host-microbe coevolution and ecological filtering based on phylogenetically structured host traits are among the best understood. However, disentangling which of these possibilities best fit a particular dataset can be difficult, as the host phylogeny and traits with phylogenetic structure are necessarily confounded. Therefore, we ran a series of trait correlation tests exploring the impacts of diet and habitat on GM diversity after controlling for the host phylogeny.

We detected significant effects of diet on GM beta diversity using UniFrac, but not star phylogeny, metrics. This discrepancy between beta diversity metrics suggests diet influences the structure of ancient microbial clades, but has no discernable effects on GM composition (after controlling for phylogeny) when all clades are weighted equally, regardless of evolutionary age. Relatedly, phylosymbiosis was often stronger using star phylogeny metrics, which likely amplify the signal of younger microbes that co-diversified with lemurs throughout the speciation process, allowing evolutionary history to override the effects of diet, which may homogenize the GM of closely-related species. Though certain species have adapted unique dietary strategies (Table S2), many aspects of the lemur diet are structured according to phylogeny, and adaptations to specific dietary guilds likely pre-date speciation events [73]. For example, cheirogaleids tend to consume a greater abundance and diversity of insects [37], while indriids and lemurids have mostly specialized on leaves and fruits, respectively (Table S2). Because each of these dietary strategies demands different digestive and metabolic processes, it stands to reason that GM diversification responds to both dietary and evolutionary divergence—which may themselves be correlated.

However, it is also possible that physiological adaptations to diet, and not diet itself, explain the significant trait correlations observed using UniFrac metrics. In a large-scale comparative study, it was found that primate GM diversity was best explained by phylogeny, even among clades that had converged on a folivorous dietary strategy [20]. Authors attributed this lack of correlation between the GM and diet to differences in gut physiology (e.g., volume, retention time, morphology), which demonstrate phylogenetic structure and, as a result, constrain GM convergence and confound comparative research—especially in studies focusing on closely-related species. Because it is difficult to isolate the effects of diet and gut physiology within our dataset, we simply conclude that diet or dietary adaptations contribute to patterns of phylosymbiosis in wild lemurs.

## Conclusions

This study shows that phylogeny plays a more important role in structuring lemur GMs than diet or habitat, though diet is also a significant factor. Our results also suggest microbes associated with host diet and speciation arose at different evolutionary time-scales, and that effects of diet have decreased in younger microbial clades. Finally, we found that closely-related allopatric species tend to harbor more similar GMs than distantly-related sympatric species. However, we suspect that niche overlap among closely-related sympatric species led to greater GM similarity than predicted from phylogenetic expectations alone, thereby weakening the phylosymbiosis signal. Future work should aim to collect and incorporate higher-resolution niche data to further parse the interplay between evolutionary and ecological factors in shaping the GMs of sympatric congeners, as this may illuminate new mechanisms facilitating co-existence of diverse lemur communities.

### Supporting data

FASTQ files are stored on NCBI under BioProject PRJNA72361. Scripts and workflows for QIIME2 and R are available at 10.6084/m9.figshare.19195571.v1. The R working directory is available at 10.6084/m9.figshare.19195574.v1.

## Supplemental material

Supplemental material

## References

[1] McFall-Ngai M, Hadfield MG, Bosch TCG, Carey HV, Domazet-Lošo T, Douglas AE (2013). Animals in a bacterial world, a new imperative for the life sciences. PNAS.

[2] Davenport ER, Sanders JG, Song SJ, Amato KR, Clark AG, Knight R (2017). The human microbiome in evolution. BMC Biol.

[3] Mazel F, Davis KM, Loudon A, Kwong WK, Groussin M, Parfrey LW (2018). Is host filtering the main driver of phylosymbiosis across the Tree of Life?. mSystems.

[4] Kohl KD (2020). Ecological and evolutionary mechanisms underlying patterns of phylosymbiosis in host-associated microbial communities. Phil Trans R Soc B..

[5] Greyson-Gaito CJ, Bartley TJ, Cottenie K, Jarvis WMC, Newman AEM, Stothart MR (2020). Into the wild: microbiome transplant studies need broader ecological reality. Proc Royal Soc B..

[6] Brooks AW, Kohl KD, Brucker RM, van Opstal EJ, Bordenstein SR. Phylosymbiosis: relationships and functional effects of microbial communities across host evolutionary history. PLOS Biol. 2016. 10.1371/journal.pbio.2000225. 10.1371/journal.pbio.2000225 PMC5115861 27861590

[7] Delsuc F, Metcalf JL, Parfrey LW, Song SJ, González A, Knight R (2014). Convergence of gut microbiomes in myrmecophagus mammals. Mol Ecol.

[8] Song SJ, Sanders JG, Delsuc F, Metcalf J, Amato K, Taylor MW (2020). Comparative analyses of vertebrate gut microbiomes reveal convergence between birds and bats. mBio.

[9] Bodawatta KH, Hird SM, Grond K, Poulsen M, Jønsson KA (2021). Avian gut microbiomes taking flight. Trends Microbiol.

[10] Egerton S, Culloty S, Whooley J, Stanton C, Ross RP. The gut microbiota of marine fish. Front Microbiol. 2018; 9. 10.3389/fmicb.2018.00873. 10.3389/fmicb.2018.00873 PMC5946678 29780377

[11] Mallott EK, Amato KR (2021). Host specificity of the gut microbiome. Nat Rev Microbiol.

[12] Ley RE, Hamady M, Lozupone C, Turnbaugh P, Ramey RR, Bircher S (2008). Evolution of mammals and their gut microbes. Science.

[13] Gogarten JF, Davies TJ, Benjamino J, Gogarten JP, Graf J, Mielke A (2018). Factors influencing bacterial microbiome composition in a wild non-human primate community in Tai National Park, Côte d’Ivoire. ISME J.

[14] Perofsky AC, Lewis RJ, Meyers LA (2019). Terrestriality and bacterial transfer: a comparative study of GMs in sympatric Malagasy mammals. ISME.

[15] Amato KR, Mallott EK, McDonald D, Dominy NJ, Goldberg T, Lambert LE (2019). Convergence of human and old world monkey gut microbiomes demonstrate the importance of human ecology over phylogeny. Genome Biol.

[16] Gomez A, Sharma AK, Mallott EK, Petrzelkova KJ, Robinson CAJ, Yeoman CJ (2019). Plasticity in the human gut microbiome defies evolutionary constraints. mSphere.

[17] Hale VL, Tan CL, Niu K, Yang Y, Knight R, Zhang Q (2018). Diet versus phylogeny: a comparison of gut microbiota in captive colobine monkey species. Microb Ecol.

[18] Ochman H, Worobey M, Kuo CH, Ndjando JBN, Peeters M, Hahn BH, et al. Evolutionary relationships of wild hominids recapitulated by gut microbial communities. PLOS Biol. 2010. 10.1371/journal.pbio.1000546. 10.1371/journal.pbio.1000546 PMC2982803 21103409

[19] McKenney EA, Maslanka M, Rodrigo A, Yoder AD. Bamboo specialists from two mammalian orders (Primates, Carnivora) share a high number of low-abundance gut microbes. Microb Ecol. 2018. 10.1007/s00248-017-1114-8. 10.1007/s00248-017-1114-8 29188302

[20] Amato KR, Sanders J, Song SJ, Nute M, Metcalf JL, Thompson LR (2019). Evolutionary trends in host physiology outweigh dietary niche in structuring primate gut microbiomes. ISME J.

[21] Bornbusch SL, Greene LK, McKenney EA, Volkoff SJ, Midani FS, Joseph G (2019). A comparative study of gut microbiomes in captive nocturnal strepsirrhines. Am J Primatol.

[22] Greene LK, Bornbusch SL, McKenney EA, Harris RL, Gorvetzian SR, Yoder AD, et al. The importance of scale in comparative microbiome research: new insights from the gut and glands of captive and wild lemurs. Am J Primatol. 2019. 10.1002/ajp.22974. 10.1002/ajp.22974 30932230

[23] Yoder AD, Nowak MD (2006). Has vicariance or dispersal been the predominant biogeographic force in Madagascar? Only time will tell. Annu Rev Ecol Evol Syst.

[24] Horvath JE, Weisrock DW, Embry SL, Fiorentino I, Balhodd JP, Kappeler P (2008). Development and application of a phylogenomic toolkit: resolving the evolutionary history of Madagascar’s lemurs. Genome Res.

[25] Herrera JP, Dávalos LM (2016). Phylogeny and divergence times of lemurs inferred with recent and ancient fossils in the tree. Syst Biol.

[26] Herrera JP (2017). Testing the adaptive radiation hypothesis for the lemurs of Madagascar. R Soc Open Sci.

[27] Herrera JP (2019). Convergent evolution in lemur environmental niches. J Biogeogr.

[28] Wright PC (1999). Lemur traits and Madagascar ecology: coping with an island environment. Am J Phys Anthropol..

[29] Greene LK, McKenney EA, O’Connell TM, Drea CM (2018). The critical role of dietary foliage in maintaining the gut microbiome and metabolome of folivorous sifakas. Sci Rep.

[30] Greene LK, Clayton JB, Rothman RS, Semel BP, Semel M, Gillespie TR (2019). Local habitat, not phylogenetic relatedness, predicts gut microbiota better within folivorous than frugivorous lemur lineages. Biol Lett.

[31] McKenney EA, O’Connell TM, Rodrigo A, Yoder AD (2018). Feeding strategy shapes gut metagenomic enrichment and functional specialization in captive lemurs. Gut Microbes.

[32] Bennett G, Malone M, Sauther ML, Cuozzo FP, White B, Nelson KE (2016). Host age, social group, and habitat type influence the gut microbiota of wild ring-tailed lemurs (*Lemur catta*). Am J Primatol.

[33] de Winter I, Umanets A, Ijdema F, Ramiro-Garcia J, van Hooft P, Heitkönig IMA (2018). Occupancy strongly influences faecal microbial composition of wild lemurs. FEMS Microbiol Ecol.

[34] Donohue ME, Absanga AE, Ralainirina J, Weisrock DW, Stumpf RM, Wright PC (2019). Extensive variability in the gut microbiome of a highly-specialized and critically endangered lemur species across sites. Am J Primatol.

[35] Fogel AT (2015). The gut microbiome of wild lemurs: a comparison of sympatric *Lemur catta* and *Propithecus verreauxi*. Folia Primatol.

[36] de Winter, Umanets A, Gort G, Nieuwland H, van Hooft P, Heitkönig IMA (2020). Effects of seasonality and previous logging on faecal helminth-microbiota associations in wild lemurs. Sci Rep.

[37] Rowe AK, Donohue ME, Clare EL, Drinkwater R, Koenig A, Ridgway ZM (2021). Exploratory analysis reveals arthropod consumption in 10 lemur species using DNA metabarcoding. Am J Primatol.

[38] Kozich JJ, Westcott SL, Baxter NT, Highlander SK, Schloss PD. Development of a dual-index sequencing strategy and curation pipeline for analyzing amplicon sequence data on the MiSeq Illumina sequencing platform. AEM. 2013. 10.1128/AEM.01043-13. 10.1128/AEM.01043-13 PMC3753973 23793624

[39] Callahan BJ, McMurdie PJ, Rosen MJ, Han AW, Johnson AJA, Holmes SP (2016). DADA2: high resolution sample inference from Illumina amplicon data. Nat Methods.

[40] Bolyen E, Rideout JR, Dillon MR, Bokulich NA, Abnet C, Al-Ghalith GA (2018). QIIME2: reproducible, interactive, scalable, and extensive microbiome data science. Nat Biotechnol..

[41] Quast C, Pruesse E, Yilmaz P, Gerken J, Schweer T, Yarza P (2013). The SILVA ribosomal RNA gene database project: improved data processing and web-based tools. Nucl Acids Res..

[42] Karstens L, Asquith M, Davin S, Fair D, Gregory WT, Wolfe AJ (2019). Controlling for contaminants in low-biomass 16S rRNA gene sequencing experiments. mSystems.

[43] Lozupone CA, Knight R (2008). Species divergence and the measurement of microbial diversity. FEMS Microbiol Rev.

[44] Sanders JG, Powell S, Kronauer DJC, Vasconcelos HL, Frederickson ME, Pierce NE (2014). Stability and phylogenetic correlation in gut microbiota: lessons from ants and apes. Mol Ecol.

[45] McMurdie PJ, Holmes S. phyloseq: an R package for reproducible interactive analysis and graphics of microbiome census data. PLOS One. 2013. 10.1371/journal.pone.0061217. 10.1371/journal.pone.0061217 PMC3632530 23630581

[46] Paliy O, Shankar V (2016). Application of multivariate statistical techniques in microbial ecology. Mol Ecol.

[47] Wickham H. ggplot2: elegant graphics for data analysis. Springer-Verlag New York. 2016.

[48] Paradis E, Schliep K (2019). ape 5.0: an environment for modern phylogenetics and evolutionary analyses in R. Bioinformatics.

[49] Revell LJ (2012). phytools: an R package for phylogenetic comparative biology (and other things). Methods Ecol Evol.

[50] Robinson DF, Foulds LR (1981). Comparison of phylogenetic trees. Math Biosci..

[51] Schliep KP (2011). phangorn: phylogenetic analysis in R. Bioinformatics.

[52] Jombart T, Balloux F, Dray S (2010). adephylo: new tools for investigating the phylogenetic signal in biological traits. Bioinformatics.

[53] Oksanen J, Blanchet FG, Kindt R, Legendre P, Minchin PR, O’Hara R, *et al*. Package “Vegan”. *Community Ecology Package*. 2016. http://CRAN.R-project.org/package=vegan.

[54] Easson CG, Thacker RW (2014). Phylogenetic signal in the community structure of host-specific microbiomes of tropical marine sponges. Front Microbiol.

[55] Blomberg SP, Garland T, Ives AR (2007). Testing for phylogenetic signal in comparative data: behavioral traits are more labile. Evolution..

[56] Pagel M (1994). Detecting correlated evolution on phylogenies: a general method for the comparative analysis of discrete characters. Proc Royal Soc B..

[57] Harmon LJ, Weird JT, Brock CD, Glor RE, Challenger W (2007). GEIGER: investigating evolutionary radiations. Bioinformatics.

[58] Phillips SJ, Anderson RP, Dudík M, Schapire RE, Blair ME (2017). Opening the black box: an open-source release of Maxent. Ecography.

[59] Hijmans RJ, Cameron SE, Parra JL, Jones PG, Jarvis A (2005). Very high resolution interpolated climate surfaces for global land areas. Int J Climatol.

[60] Orme D, Freckleton R, Thomas G, Petzoldt T, Fritz S, Isaac N, et al. CAPER: comparative analyses of phylogenetics and evolution in R. 2012. http://cran.r-project.org/package=caper.

[61] Groussin M, Mazel F, Sanders JG, Smillie CS, Lavergne S, Thuiller W (2017). Unraveling the processes shaping mammalian GMs over evolutionary time. Nat Commun.

[62] Knowles SCL, Eccles RM, Baltrūnaitė L (2019). Species identity dominates over environment in shaping the microbiota of small mammals. Ecol Lett.

[63] Kohl KD, Varner J, Wilkening JL, Dearing MD (2018). Gut microbial communities of American pikas (*Ochotona princeps*): evidence for phylosymbiosis and adaptations to novel diets. J Anim Ecol.

[64] Kohl KD, Dearing MD, Bordenstein SR (2018). Microbial communities exhibit host species distinguishability and phylosymbiosis along the length of the gastrointestinal tract. Mol Ecol.

[65] Weinstein SB, Martínez-Mota R, Stapleton TE, Klure DM, Greeenhalgh R, Orr TJ, et al. Microbiome stability and structure is governed by host phylogeny over diet and geography in woodrats (*Neotoma* spp.). PNAS. 118:e2108787118. 10.1073/pnas.2108787118. 10.1073/pnas.2108787118 PMC8617456 34799446

[66] Grond K, Bell KC, Demboski JR, Santos M, Sullivan JM, Hird SM (2020). No evidence for phylosymbiosis in western chipmunk species. FEMS Microbiol Ecol.

[67] Perofsky AC, Lewis RJ, Adondano LA, Di Fiore A, Meyers LA (2017). Hierarchical social networks shape gut microbial composition in wild Verreaux’s sifaka. Proc R Soc B..

[68] Raulo A, Ruokolainen L, Lane A, Amato K, Knight R, Leigh S (2017). Social behavior and gut microbiota in red-bellied lemurs (*Eulemur rubriventer*): in search of the role of immunity in the evolution of sociality. J Anim Ecol.

[69] Overdorff DJ (1993). Similarities, differences, and seasonal patterns in the diets of *Eulemur rubriventer* and *Eulemur fulvus rufus* in the Ranomafana National Park, Madagascar. Int J Primatol.

[70] Johnson SE. Ecology and speciation in brown lemurs: white-collared lemur (*Eulemur albocollaris*) and hybrids (*Eulemur albocollaris* x *Eulemur fulvus rufus*) in southeastern Madagascar. 2002. PhD Dissertation. The University of Texas at Austin.

[71] Wyner YM, Johnson SE, Stumpf RM, DeSalle R (2002). Genetic assessment of a white-collared x red-fronted lemur hybrid zone at Andringitra, Madagascar. Am J Primatol.

[72] Hladik CM, Charles-Dominique P, Petter JJ. Feeding strategies of five nocturnal prosimians in the dry forest of the west coast of Madagascar. In: Charles-Dominique P, Cooper HM, Hladik A, Pages E, Pariente GF, Petter-Rousseaux A, Schilling A (eds). Nocturnal Malagasy primates: ecology, physiology, and behavior. Academic Press, New York, NY. 1980. pp 41-73. 10.1016/B978-0-12-169350-3.50007-1.

[73] Perelman P, Johnson WE, Roos C, Seuánez HN, Horvath JE, Moreira MAM, et al. A molecular phylogeny of living primates. PLOS Genet. 2011. 10.1371/journal.pgen.1001342. 10.1371/journal.pgen.1001342 PMC3060065 21436896

