# Supplementary material for: Significant effects of host dietary guild and phylogeny in wild lemur gut microbiomes
Source: ISME Commun. 2022 Apr 8;2:33. doi: 10.1038/s43705-022-00115-6 (PMC9723590; doi:10.1038/s43705-022-00115-6)
Supplement: Supplementary file 1 — Supplemental material [file 43705_2022_115_MOESM1_ESM.pdf]

Supporting Information

Methods

Constructing ecological niche models (ENMs)

To construct ENMs, we downloaded 19 WorldClim (Hijmans et al. 2005) layers in 2.5-min arc resolution. Autocorrelation analyses in ENMTools (v1.3; Warren et al. 2010) identified seven collinear variables with Pearson correlations > 0.90, which were removed from downstream analyses. The 12 layers retained were: mean temperature of coldest quarter, annual precipitation, precipitation of wettest month, precipitation seasonality, precipitation of wettest quarter, precipitation of driest quarter, precipitation of warmest quarter, isothermality, temperature seasonality, maximum temperature of warmest month, minimum temperature of coldest month, and temperature annual range. Layers were clipped in QGIS v3.6.

ENMs were constructed in Maxent v. 3.4.1 (Phillips et al. 2017) using 75% of the occurrence data for model training and the remaining 25% for model testing. We parameterized habitat suitability models using five replicates, 500 maximum iterations, and a convergence threshold of 0.00001. Model accuracy was evaluated by area under the curve (AUC), a test statistic that measures the ability of each model to discriminate between presence and absence sites, thus correctly selecting areas in terms of their relative importance for a given species. AUC values range from 0 to 1; typically, values higher than 0.8 are considered informative (Hosmer & Lemeshow 2000).

As an additional confirmation that our ENMs accurately represented the ecology of each species, we conducted pairwise comparisons of niche overlap using the D-metric in ENMTools. This test statistic ranges from 0 (no niche overlap) to 1 (identical niches; see Warren et al. 2008).

Figures

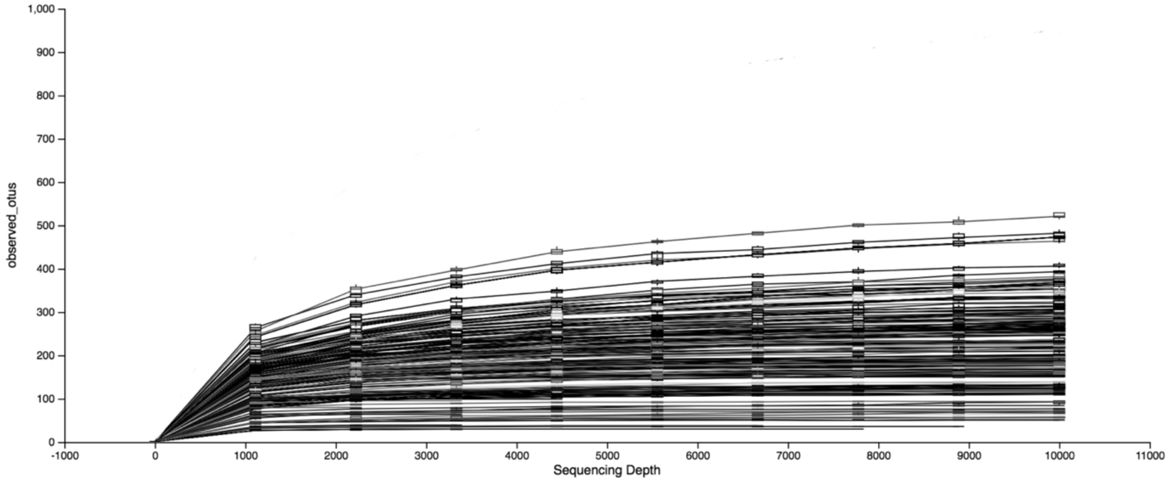

Fig. S1: Rarefaction plot of alpha diversity (metric: observed OTUs).

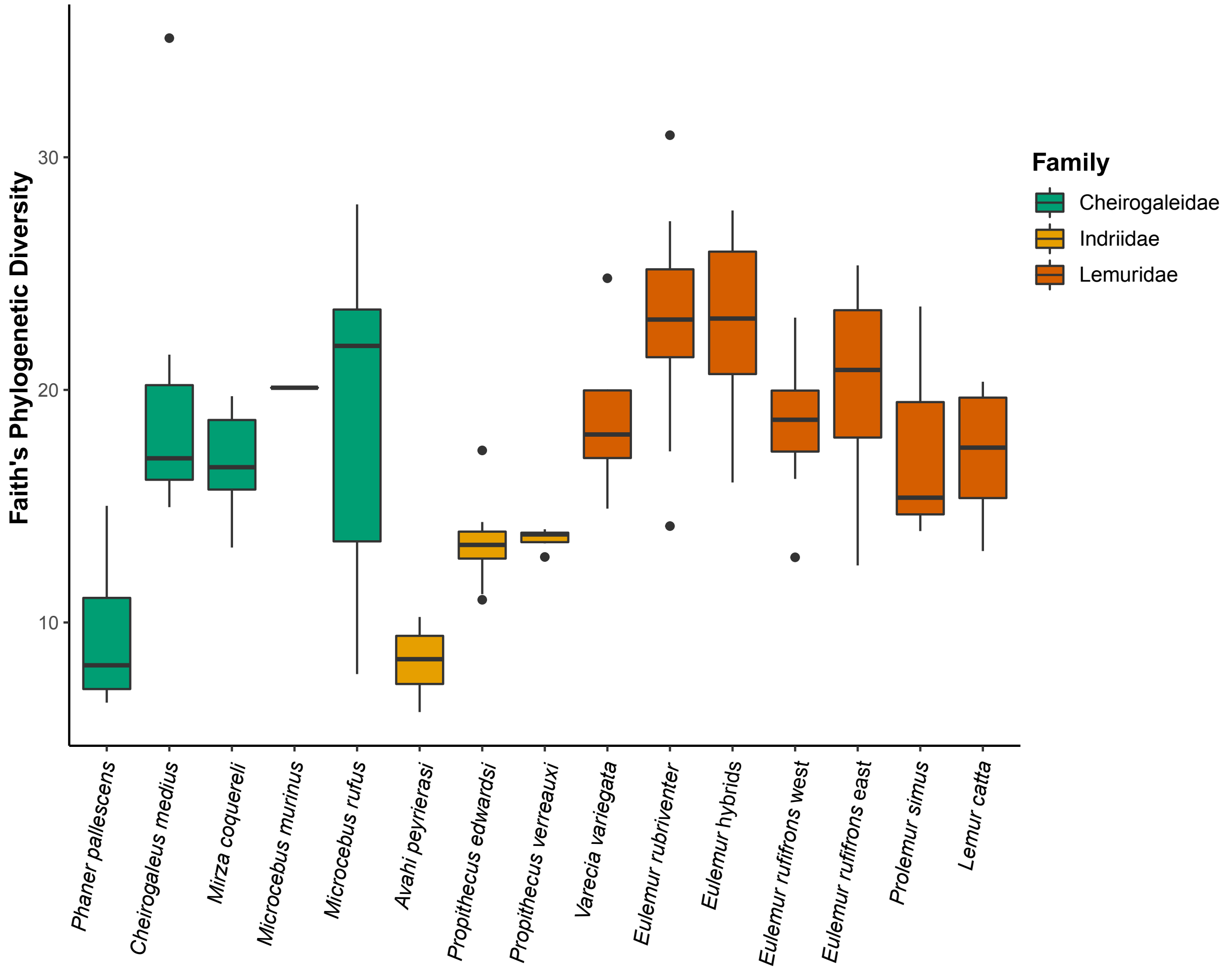

Fig. S2: Boxplot showing differences in alpha diversity (Faith's PD) across species and families.

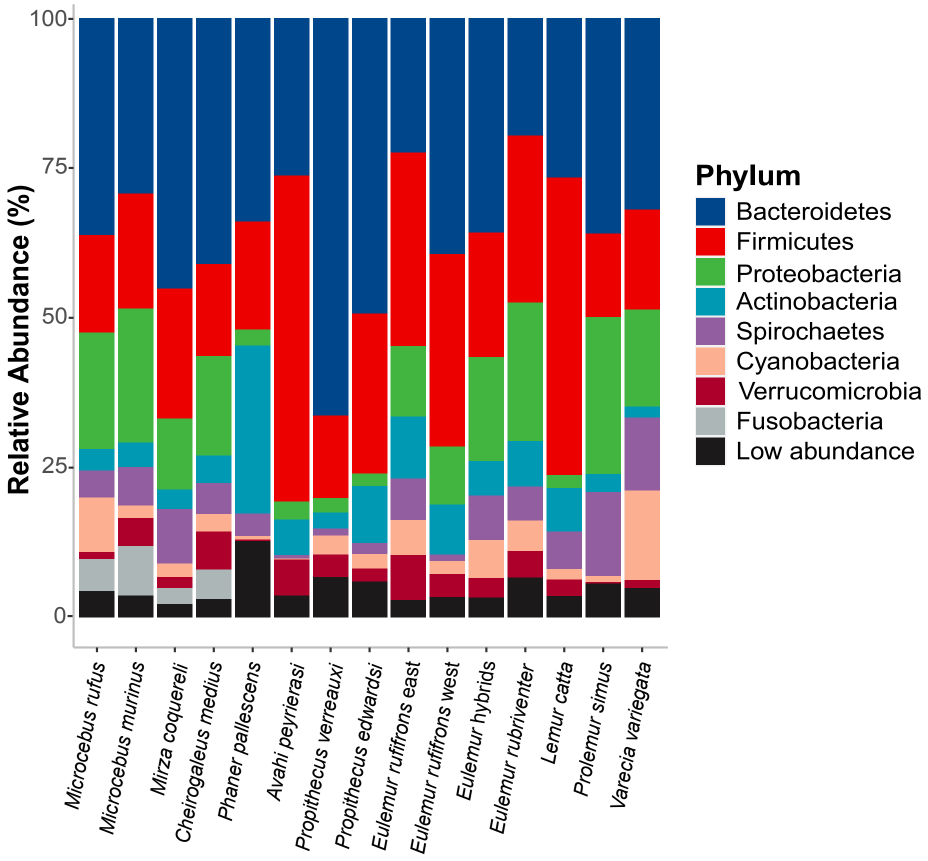

Fig. S3: Barplot showing relative abundance of microbial phyla, grouped by species.

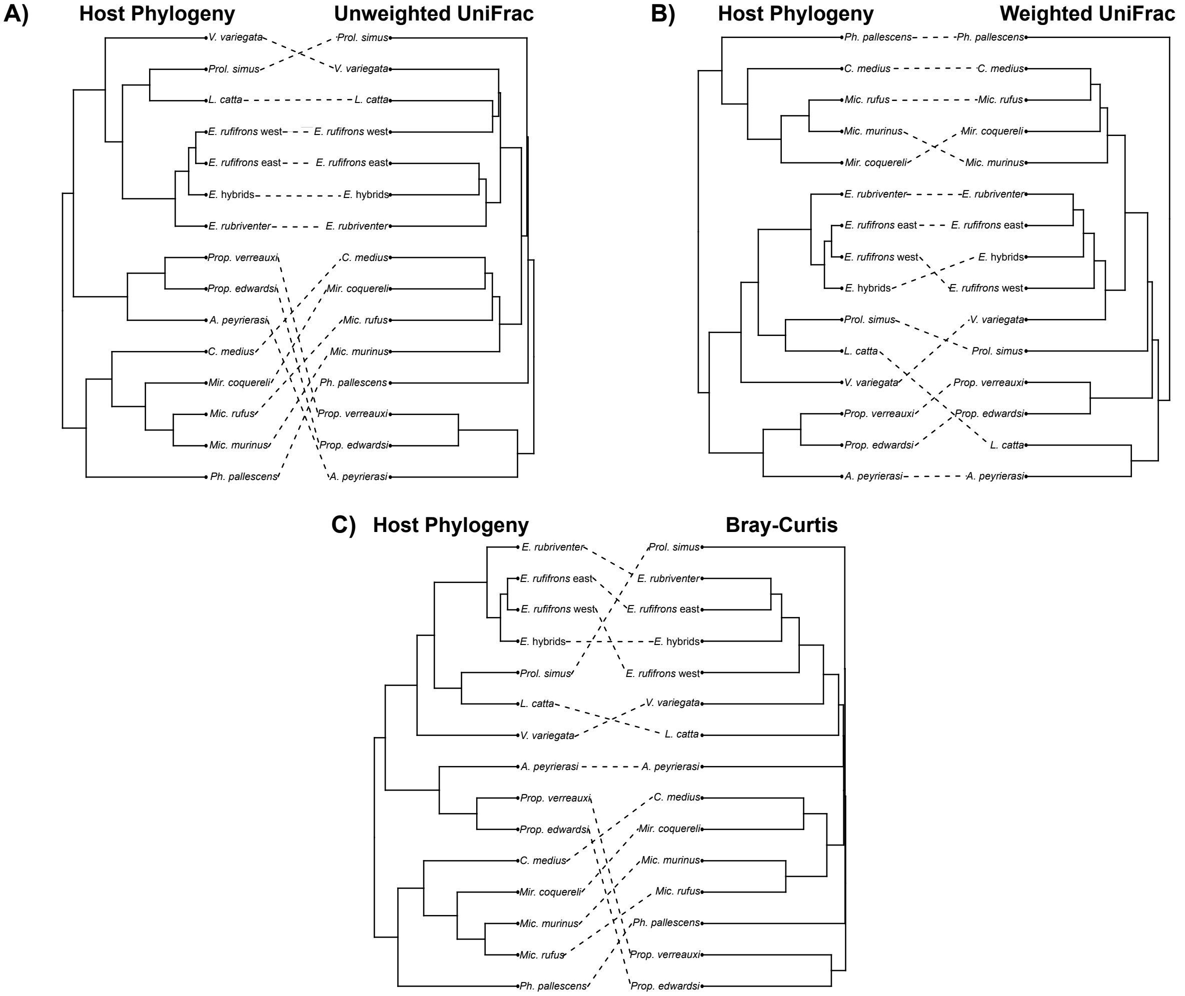

Fig. S4: Co-dendrogram comparison of branching order between the host phylogeny (left) and host-aggregated UPGMA trees (**A**: unweighted UniFrac, **B**: weighted UniFrac, **C**: Bray-Curtis dissimilarity). Dashed lines connect tip labels between trees.

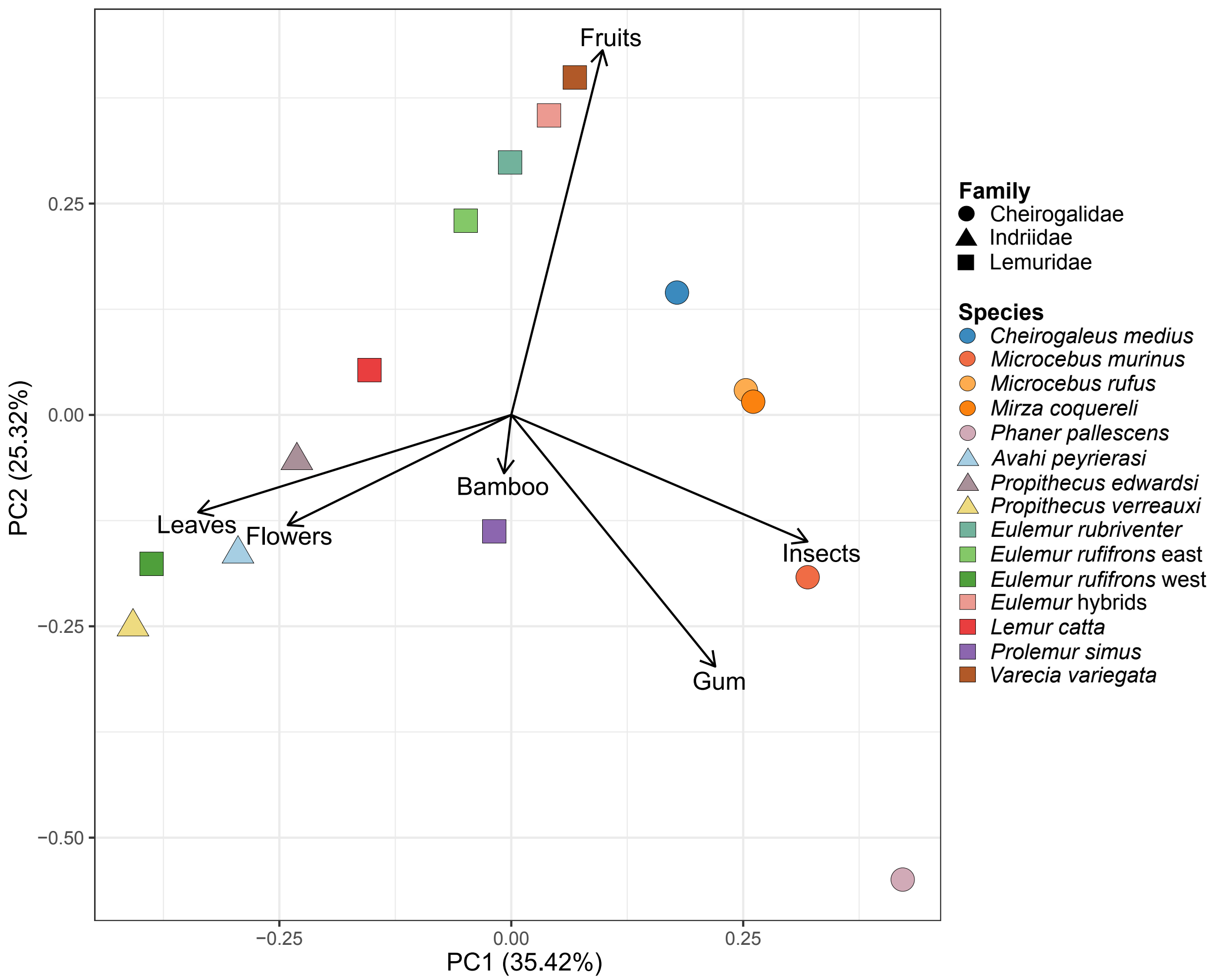

Fig. S5: PCA of lemur dietary guild, measured using the percent of total feeding time species were observed consuming each major food group in representative feeding behavior studies (see Table S2). Data points are coded by family (shape) and species (color).

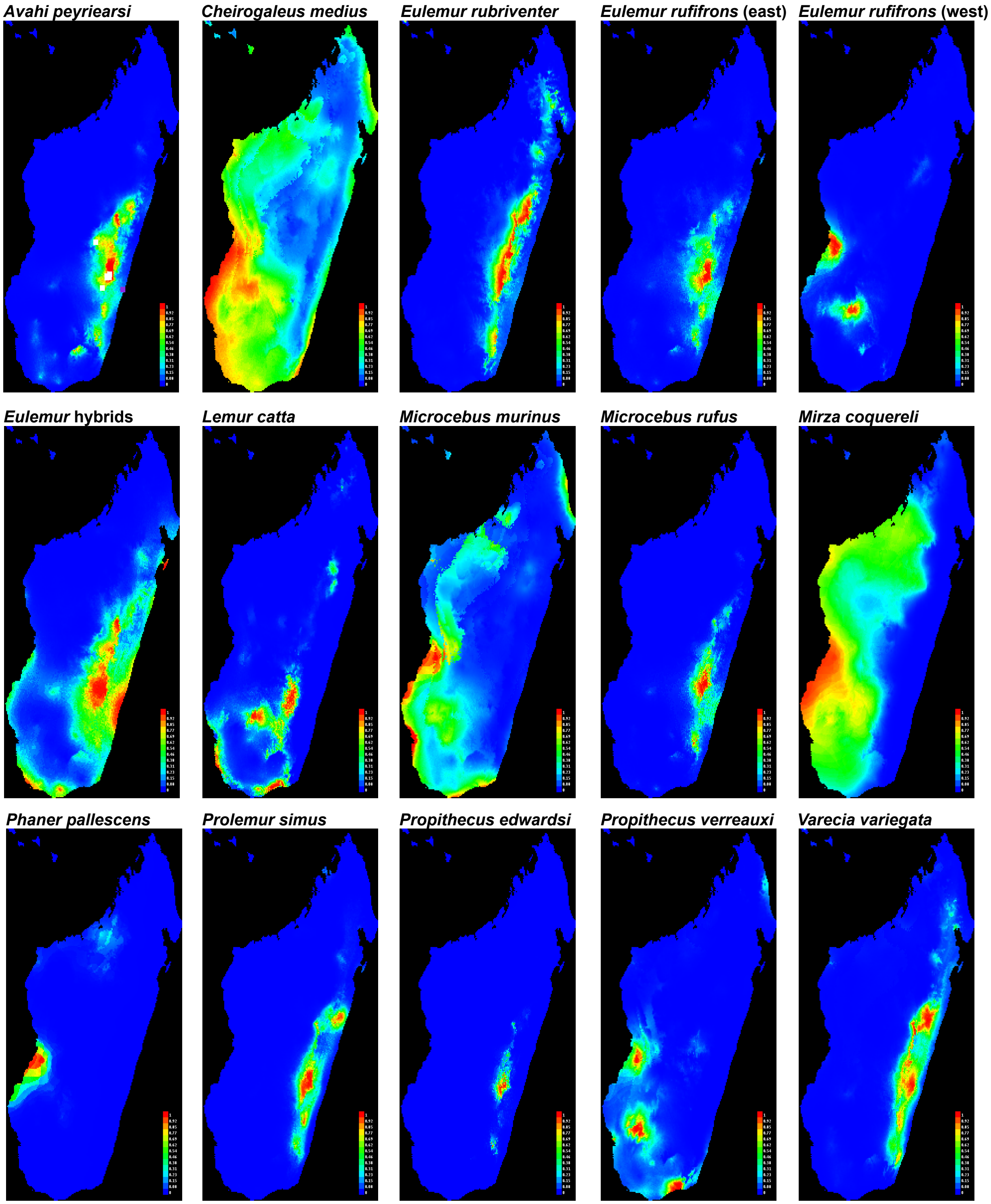

Fig. S6: Ecological niche models (ENMs) for each study species. Areas shaded red represent the highest habitat suitability, while blue areas represent the lowest. Areas of intermediate suitability are shaded orange (higher suitability), yellow, or green (lower suitability).

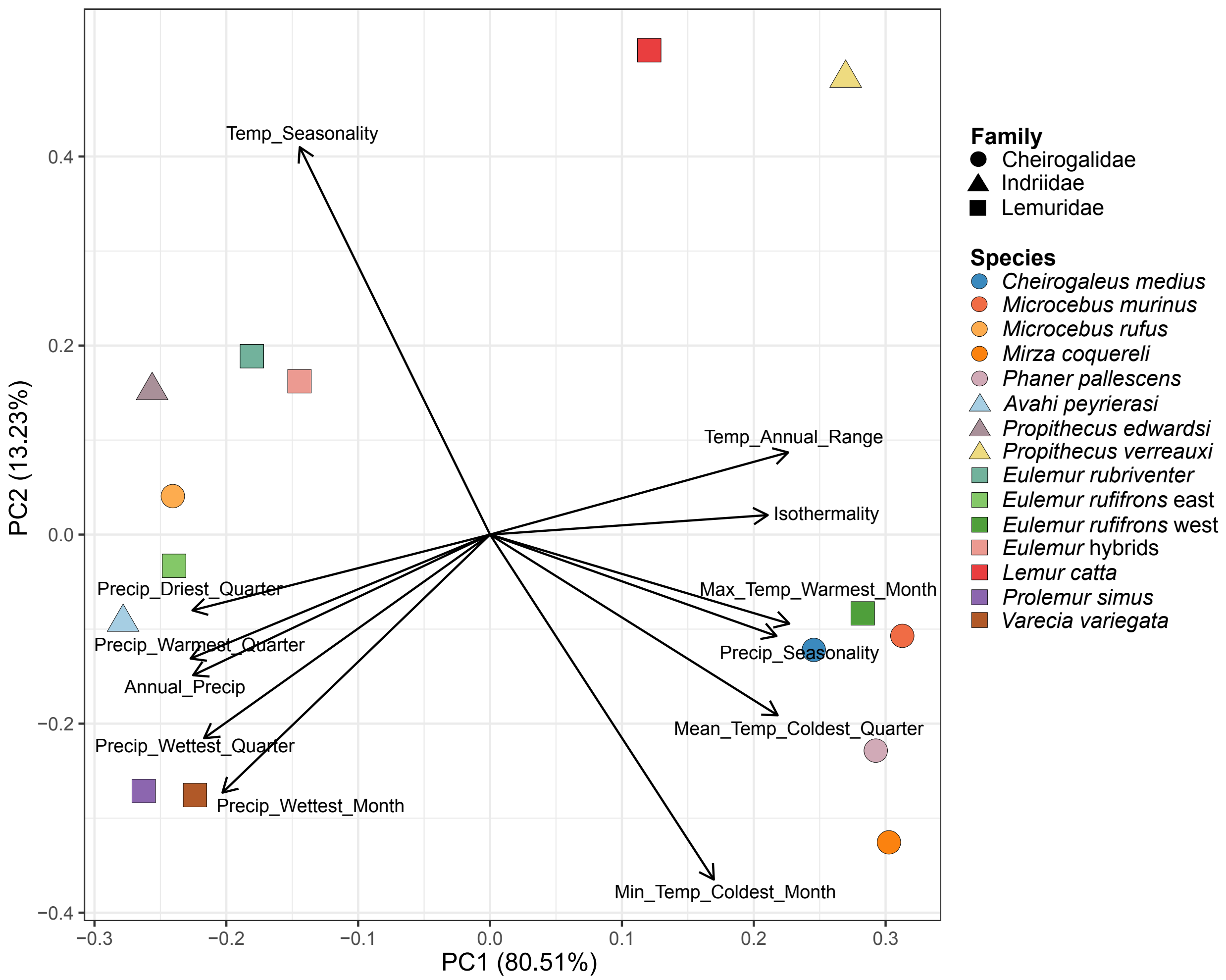

Fig. S7: PCA of the climatic factors influencing habitat suitability for each lemur species, as determined using ENMs (see Fig. S6). Data points are coded by family (shape) and species (color).

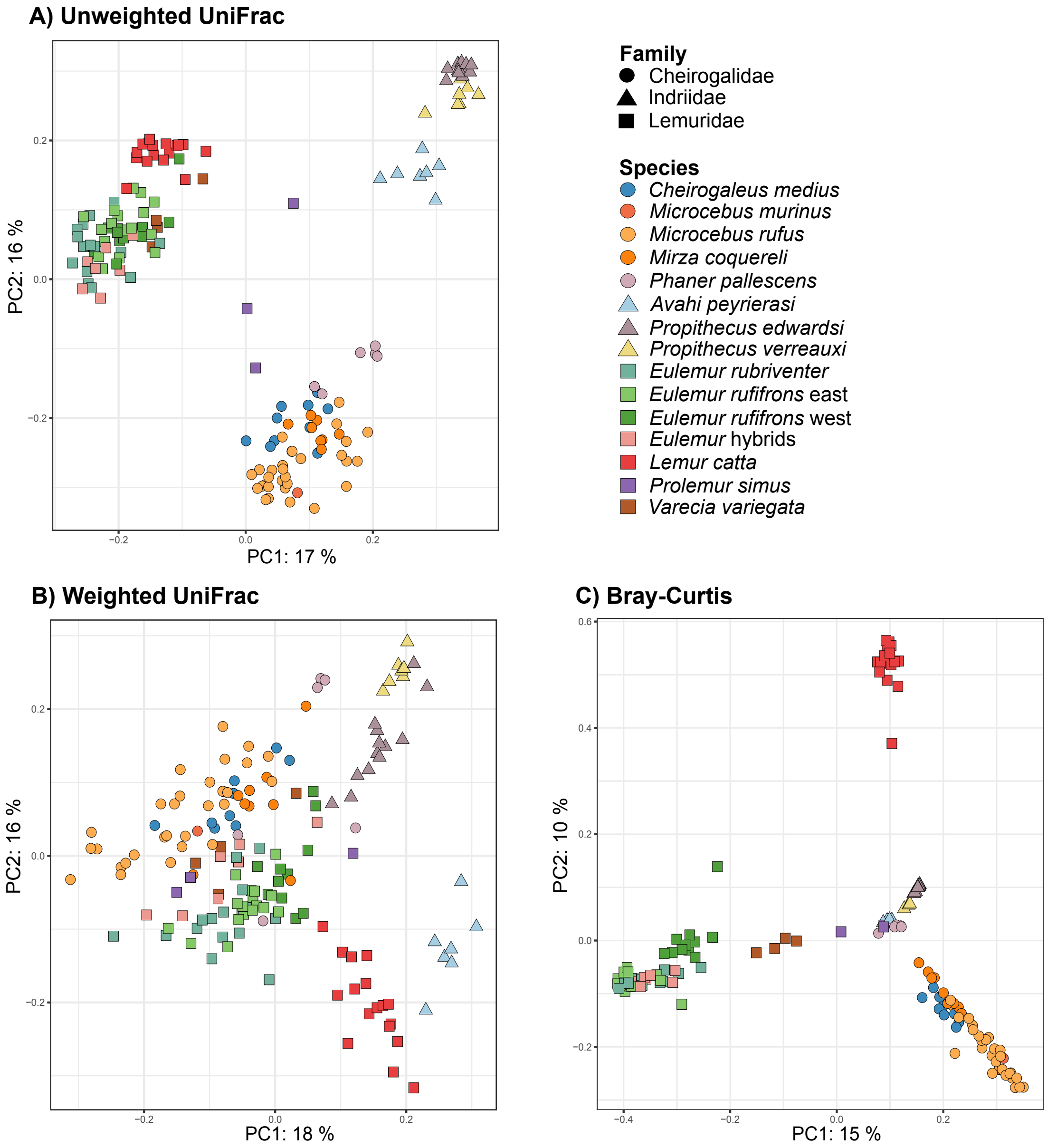

Fig. S8: PCoA plots of GM diversity (**A**: unweighted UniFrac, **B**: weighted UniFrac, **C**: Bray-Curtis dissimilarity) coded by host family (shape) and species (color).

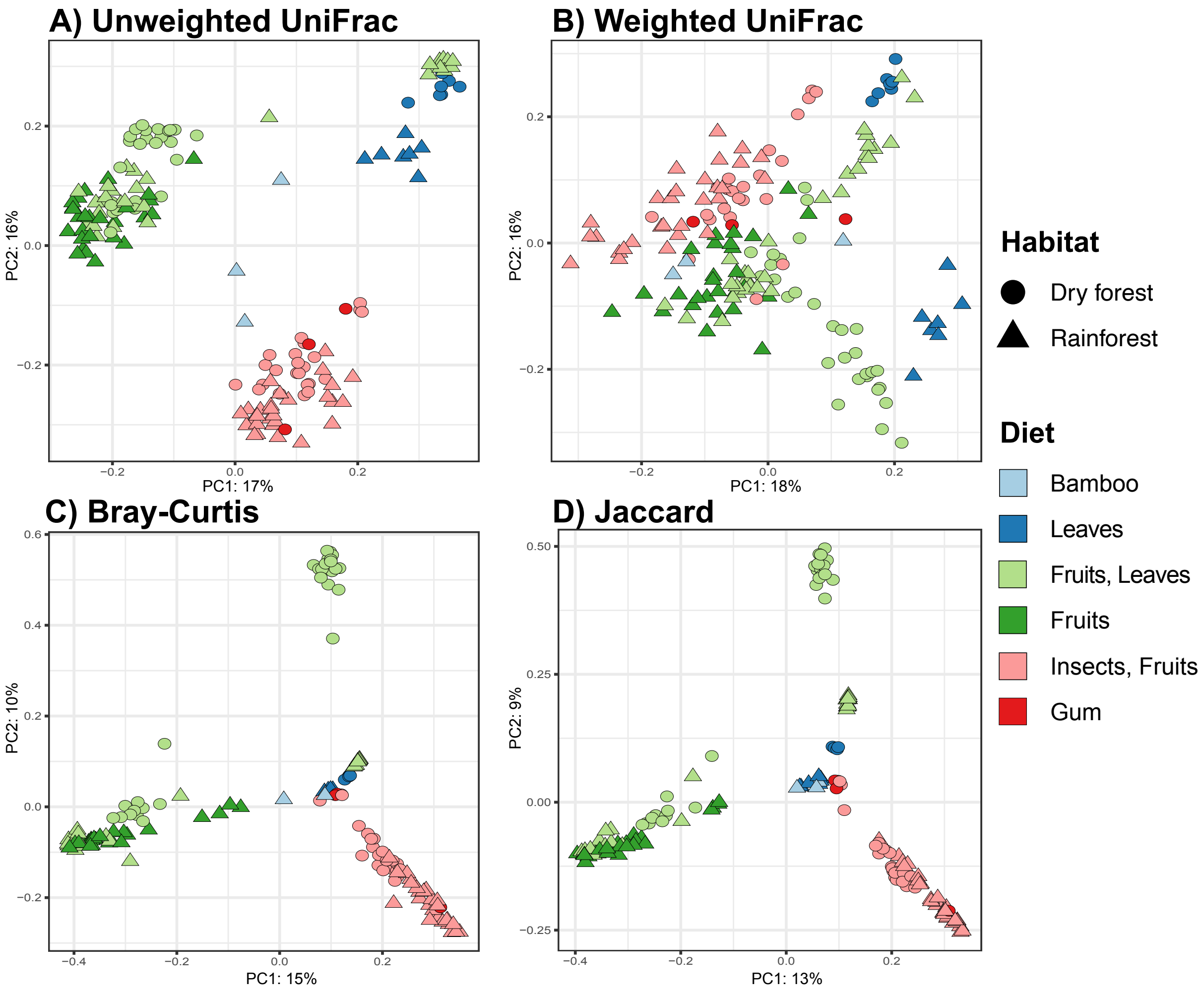

Fig. S9: PCoA plots of GM diversity (**A**: unweighted UniFrac, **B**: weighted UniFrac, **C**: Bray-Curtis dissimilarity, D: Jaccard) coded by habitat of the sampling locality (shape) and host species diet (color).

Supporting Tables

Table S1: ASVs and number of sequences found in PCR negative controls.

**ASV**	**# sequences**
Bacteria; Acidobacteria; Acidobacteriia; Acidobacteriales; Acidobacteriaceae (Subgroup 1); Granulicella	6
Bacteria; Acidobacteria; Acidobacteriia; Subgroup 2; uncultured Acidobacteria bacterium	1
Bacteria; Acidobacteria; Blastocatellia (Subgroup 4); Blastocatellales; Blastocatellaceae; Blastocatella; uncultured bacterium gp4	6
Bacteria; Acidobacteria; Subgroup 6	43
Bacteria; Actinobacteria; Acidimicrobiia; IMCC26256	2
Bacteria; Actinobacteria; Acidimicrobiia; Microtrichales; Ilumatobacteraceae; uncultured	1
Bacteria; Actinobacteria; Actinobacteria; Actinomycetales; Actinomycetaceae; Actinomyces	17
Bacteria; Actinobacteria; Actinobacteria; Bifidobacteriales; Bifidobacteriaceae; Bifidobacterium	26
Bacteria; Actinobacteria; Actinobacteria; Corynebacteriales; Corynebacteriaceae; Corynebacterium 1; Corynebacterium massiliense DSM 45435	8
Bacteria; Actinobacteria; Actinobacteria; Corynebacteriales; Corynebacteriaceae; Corynebacterium 1	750
Bacteria; Actinobacteria; Actinobacteria; Corynebacteriales; Corynebacteriaceae; Lawsonella	176
Bacteria; Actinobacteria; Actinobacteria; Corynebacteriales; Mycobacteriaceae; Mycobacterium	4
Bacteria; Actinobacteria; Actinobacteria; Corynebacteriales; Nocardiaceae; Rhodococcus	28
Bacteria; Actinobacteria; Actinobacteria; Corynebacteriales; Nocardiaceae; Williamsia	1
Bacteria; Actinobacteria; Actinobacteria; Frankiales; Geodermatophilaceae; Geodermatophilus	26
Bacteria; Actinobacteria; Actinobacteria; Kineosporiales; Kineosporiaceae; Quadrisphaera; Quadrisphaera	31
Bacteria; Actinobacteria; Actinobacteria; Kineosporiales; Kineosporiacea	3
Bacteria; Actinobacteria; Actinobacteria; Micrococcales; Cellulomonadaceae; Pseudactinotalea	34
Bacteria; Actinobacteria; Actinobacteria; Micrococcales; Dermabacteraceae; Brachybacterium	110
Bacteria; Actinobacteria; Actinobacteria; Micrococcales; Intrasporangiaceae	19
Bacteria; Actinobacteria; Actinobacteria; Micrococcales; Microbacteriaceae; Agromyces	5
Bacteria; Actinobacteria; Actinobacteria; Micrococcales; Microbacteriaceae; Microbacterium	8
Bacteria; Actinobacteria; Actinobacteria; Micrococcales; Microbacteriaceae	7
Bacteria; Actinobacteria; Actinobacteria; Micrococcales; Micrococcaceae; Glutamicibacter	41
Bacteria; Actinobacteria; Actinobacteria; Micrococcales; Micrococcaceae; Kocuria	25
Bacteria; Actinobacteria; Actinobacteria; Micrococcales; Micrococcaceae; Micrococcus	4

Bacteria; Actinobacteria; Actinobacteria; Micrococcales; Micrococcaceae; Nesterenkonia	4
Bacteria; Actinobacteria; Actinobacteria; Micromonosporales; Micromonosporaceae	3
Bacteria; Actinobacteria; Actinobacteria; Propionibacteriales; Nocardioidaceae; Aeromicrobium	2
Bacteria; Actinobacteria; Actinobacteria; Propionibacteriales; Nocardioidaceae; Nocardioides; Nocardioides sp. KV-833	17
Bacteria; Actinobacteria; Actinobacteria; Propionibacteriales; Nocardioidaceae; Nocardioides	45
Bacteria; Actinobacteria; Actinobacteria; Propionibacteriales; Propionibacteriaceae; Cutibacterium	336
Bacteria; Actinobacteria; Actinobacteria; Propionibacteriales; Propionibacteriaceae; Friedmanniella	5
Bacteria; Actinobacteria; Actinobacteria	2
Bacteria; Actinobacteria; Coriobacteriia; Coriobacteriales; Atopobiaceae; Olsenella	20
Bacteria; Actinobacteria; Coriobacteriia; Coriobacteriales; Eggerthellaceae; uncultured	1
Bacteria; Actinobacteria; Thermoleophilia; Solirubrobacterales; Solirubrobacteraceae; Solirubrobacter	4
Bacteria; Armatimonadetes; Armatimonadia; Armatimonadales; uncultured bacterium; ;	5
Bacteria; Bacteroidetes; Bacteroidia; Bacteroidales; Muribaculaceae; uncultured bacterium;	1
Bacteria; Bacteroidetes; Bacteroidia; Bacteroidales; Muribaculaceae	3
Bacteria; Bacteroidetes; Bacteroidia; Bacteroidales; Porphyromonadaceae; Porphyromonas; Porphyromonas cangingivalis	24
Bacteria; Bacteroidetes; Bacteroidia; Bacteroidales; Prevotellaceae; Alloprevotella; uncultured bacterium	8
Bacteria; Bacteroidetes; Bacteroidia; Bacteroidales; Prevotellaceae; Alloprevotella	19
Bacteria; Bacteroidetes; Bacteroidia; Bacteroidales; Prevotellaceae; Prevotella 6; uncultured bacterium	15
Bacteria; Bacteroidetes; Bacteroidia; Bacteroidales; Prevotellaceae; Prevotella	21
Bacteria; Bacteroidetes; Bacteroidia; Bacteroidales; Prevotellaceae; Prevotellaceae UCG-001; uncultured bacterium	1
Bacteria; Bacteroidetes; Bacteroidia; Bacteroidales; Prevotellaceae; Prevotellaceae UCG-001	8
Bacteria; Bacteroidetes; Bacteroidia; Bacteroidales; Prevotellaceae	88
Bacteria; Bacteroidetes; Bacteroidia; Bacteroidales	39
Bacteria; Bacteroidetes; Bacteroidia; Chitinophagales; Chitinophagaceae; Haoranjiania; uncultured bacterium	72
Bacteria; Bacteroidetes; Bacteroidia; Cytophagales; Hymenobacteraceae; Hymenobacter; uncultured Bacteroidetes bacterium	21
Bacteria; Bacteroidetes; Bacteroidia; Cytophagales; Hymenobacteraceae; Hymenobacter	70
Bacteria; Bacteroidetes; Bacteroidia; Cytophagales; Spirosomaceae; Dyadobacter	98
Bacteria; Bacteroidetes; Bacteroidia; Flavobacteriales; Flavobacteriaceae; Flavobacterium	47
Bacteria; Bacteroidetes; Bacteroidia; Flavobacteriales; Weeksellaceae; Chryseobacterium	3
Bacteria; Bacteroidetes; Bacteroidia; Sphingobacteriales; AKYH767	98
Bacteria; Bacteroidetes; Bacteroidia; Sphingobacteriales; Sphingobacteriaceae; Pedobacter	7

Bacteria; Bacteroidetes; Bacteroidia; Sphingobacteriales; Sphingobacteriaceae; Sphingobacterium; Sphingobacterium bambusae	7
Bacteria; Bacteroidetes; Bacteroidia; Sphingobacteriales; Sphingobacteriaceae; Sphingobacterium; Sphingobacterium shayense	615
Bacteria; Bacteroidetes; Bacteroidia; Sphingobacteriales; Sphingobacteriaceae; Sphingobacterium; Sphingobacterium sp. 23D10-4-9	40
Bacteria; Bacteroidetes; Rhodothermia; Rhodothermales; Rhodothermaceae; Rubrivirga; uncultured bacterium	5
Bacteria; Chloroflexi; Anaerolineae; SBR1031; A4b; metagenome;	8
Bacteria; Cyanobacteria; Melainabacteria; Gastranaerophilales; uncultured rumen bacterium; ;	2
Bacteria; Cyanobacteria; Melainabacteria; Gastranaerophilales	70
Bacteria; Cyanobacteria; Oxyphotobacteria; Chloroplast	608
Bacteria; Cyanobacteria; Oxyphotobacteria; Synechococcales; Cyanobiaceae; Cyanobium PCC-6307	1
Bacteria; Deinococcus-Thermus; Deinococci; Deinococcales; Deinococcaceae; Deinococcus; Deinococcus sp. 2A5	1
Bacteria; Epsilonbacteraeota; Campylobacteria; Campylobacterales; Campylobacteraceae; Campylobacter	1
Bacteria; Fibrobacteres; Fibrobacteria; Fibrobacterales; Fibrobacteraceae; Fibrobacter	1
Bacteria; Firmicutes; Bacilli; Bacillales; Bacillaceae; Bacillus	14
Bacteria; Firmicutes; Bacilli; Bacillales; Family XI; Gemella	16
Bacteria; Firmicutes; Bacilli; Bacillales; Staphylococcaceae; Staphylococcus	1052
Bacteria; Firmicutes; Bacilli; Bacillales; Staphylococcaceae	1282
Bacteria; Firmicutes; Bacilli; Bacillales	42
Bacteria; Firmicutes; Bacilli; Lactobacillales; Aerococcaceae; Aerococcus	7
Bacteria; Firmicutes; Bacilli; Lactobacillales; Lactobacillaceae; Lactobacillus; Lactobacillus iners AB-1	15
Bacteria; Firmicutes; Bacilli; Lactobacillales; Lactobacillaceae; Lactobacillus	17
Bacteria; Firmicutes; Bacilli; Lactobacillales; Leuconostocaceae; Leuconostoc	18
Bacteria; Firmicutes; Bacilli; Lactobacillales; Leuconostocaceae; Weissella	9
Bacteria; Firmicutes; Bacilli; Lactobacillales; Streptococcaceae; Streptococcus; Streptococcus thermophilus TH1435	92
Bacteria; Firmicutes; Bacilli; Lactobacillales; Streptococcaceae; Streptococcus	119
Bacteria; Firmicutes; Clostridia; Clostridiales; Clostridiales vadinBB60 group	1
Bacteria; Firmicutes; Clostridia; Clostridiales; Family XI; Anaerococcus; uncultured bacterium	18
Bacteria; Firmicutes; Clostridia; Clostridiales; Family XI; Anaerococcus	35
Bacteria; Firmicutes; Clostridia; Clostridiales; Family XI; Finegoldia	9
Bacteria; Firmicutes; Clostridia; Clostridiales; Family XI; Helcococcus; uncultured bacterium	81

Bacteria; Firmicutes; Clostridia; Clostridiales; Lachnospiraceae; GCA-900066575	22
Bacteria; Firmicutes; Clostridia; Clostridiales; Lachnospiraceae; Moryella	3
Bacteria; Firmicutes; Clostridia; Clostridiales; Lachnospiraceae; Roseburia	86
Bacteria; Firmicutes; Clostridia; Clostridiales; Lachnospiraceae	151
Bacteria; Firmicutes; Clostridia; Clostridiales; Ruminococcaceae; Ruminiclostridium 9	19
Bacteria; Firmicutes; Clostridia; Clostridiales; Ruminococcaceae; Ruminococcaceae UCG-014	155
Bacteria; Firmicutes; Erysipelotrichia; Erysipelotrichales; Erysipelotrichaceae; Erysipelotrichaceae UCG-004; uncultured bacterium	61
Bacteria; Firmicutes; Erysipelotrichia; Erysipelotrichales; Erysipelotrichaceae; Erysipelotrichaceae UCG-004; unidentified rumen bacterium RFN74	16
Bacteria; Firmicutes; Negativicutes; Selenomonadales; Veillonellaceae; Veillonella; unidentified	68
Bacteria; Firmicutes; Negativicutes; Selenomonadales; Veillonellaceae; Veillonella	22
Bacteria; Fusobacteria; Fusobacteriia; Fusobacteriales; Fusobacteriaceae; Fusobacterium	91
Bacteria; Fusobacteria; Fusobacteriia; Fusobacteriales; Leptotrichiaceae; Leptotrichia	55
Bacteria; Fusobacteria; Fusobacteriia; Fusobacteriales; Leptotrichiaceae	1
Bacteria; Kiritimatiellaeota; Kiritimatiellae; WCHB1-41	38
Bacteria; Nitrospirae; Nitrospira; Nitrospirales; Nitrospiraceae; Nitrospira	26
Bacteria; Planctomycetes; Planctomycetacia; Gemmatales; Gemmataceae; Zavarzinella	3
Bacteria; Planctomycetes; Planctomycetacia; Isosphaerales; Isosphaeraceae; Singulisphaera	2
Bacteria; Planctomycetes; Planctomycetacia; Pirellulales; Pirellulaceae; Pir4 lineage	2
Bacteria; Proteobacteria; Alphaproteobacteria; Acetobacterales; Acetobacteraceae; Roseomonas	4
Bacteria; Proteobacteria; Alphaproteobacteria; Caulobacterales; Caulobacteraceae; Brevundimonas	173
Bacteria; Proteobacteria; Alphaproteobacteria; Caulobacterales; Caulobacteraceae; Caulobacter	4
Bacteria; Proteobacteria; Alphaproteobacteria; Rhizobiales; Beijerinckiaceae; Methylobacterium	393
Bacteria; Proteobacteria; Alphaproteobacteria; Rhizobiales; Beijerinckiaceae; uncultured; Methylobacterium sp. AG46	35
Bacteria; Proteobacteria; Alphaproteobacteria; Rhizobiales; Beijerinckiaceae; uncultured; bacterium	3
Bacteria; Proteobacteria; Alphaproteobacteria; Rhizobiales; Beijerinckiaceae	6
Bacteria; Proteobacteria; Alphaproteobacteria; Rhizobiales; Devosiaceae; Devosia	56
Bacteria; Proteobacteria; Alphaproteobacteria; Rhizobiales; Rhizobiaceae; Allorhizobium-Neorhizobium-Pararhizobium-Rhizobium	261
Bacteria; Proteobacteria; Alphaproteobacteria; Rhizobiales; Rhizobiaceae; Aureimonas	39
Bacteria; Proteobacteria; Alphaproteobacteria; Rhizobiales; Rhizobiaceae; Mesorhizobium	86

Bacteria; Proteobacteria; Alphaproteobacteria; Rhizobiales; Rhizobiaceae; Mesorhizobium	90
Bacteria; Proteobacteria; Alphaproteobacteria; Rhizobiales; Rhizobiaceae	3
Bacteria; Proteobacteria; Alphaproteobacteria; Rhizobiales; Xanthobacteraceae	67
Bacteria; Proteobacteria; Alphaproteobacteria; Rhodobacterales; Rhodobacteraceae; Rubellimicrobium; uncultured bacterium	6
Bacteria; Proteobacteria; Alphaproteobacteria; Rhodobacterales; Rhodobacteraceae; Rubellimicrobium	5
Bacteria; Proteobacteria; Alphaproteobacteria; Rickettsiales; Mitochondria; Zasmidium cellare;	36
Bacteria; Proteobacteria; Alphaproteobacteria; Rickettsiales; Mitochondria; uncultured bacterium;	19
Bacteria; Proteobacteria; Alphaproteobacteria; Rickettsiales; Mitochondria	39
Bacteria; Proteobacteria; Alphaproteobacteria; Sphingomonadales; Sphingomonadaceae; Altererythrobacter	1
Bacteria; Proteobacteria; Alphaproteobacteria; Sphingomonadales; Sphingomonadaceae; Novosphingobium	78
Bacteria; Proteobacteria; Alphaproteobacteria; Sphingomonadales; Sphingomonadaceae; Sphingomonas	19769
Bacteria; Proteobacteria; Deltaproteobacteria; Bdellovibrionales; Bdellovibrionaceae; Bdellovibrio; metagenome	4
Bacteria; Proteobacteria; Deltaproteobacteria; Bdellovibrionales; Bdellovibrionaceae; Bdellovibrio; uncultured bacterium	48
Bacteria; Proteobacteria; Deltaproteobacteria; Desulfuromonadales; Geobacteraceae; Geoalkalibacter; uncultured bacterium	36
Bacteria; Proteobacteria; Gammaproteobacteria; Aeromonadales; Succinivibrionaceae	17
Bacteria; Proteobacteria; Gammaproteobacteria; Alteromonadales; Shewanellaceae; Shewanella	17
Bacteria; Proteobacteria; Gammaproteobacteria; Betaproteobacteriales; Burkholderiaceae; Janthinobacterium	68
Bacteria; Proteobacteria; Gammaproteobacteria; Betaproteobacteriales; Burkholderiaceae; Massilia	361
Bacteria; Proteobacteria; Gammaproteobacteria; Betaproteobacteriales; Burkholderiaceae; Pigmentiphaga	6
Bacteria; Proteobacteria; Gammaproteobacteria; Betaproteobacteriales; Burkholderiaceae; Ralstonia	29
Bacteria; Proteobacteria; Gammaproteobacteria; Betaproteobacteriales; Burkholderiaceae	289
Bacteria; Proteobacteria; Gammaproteobacteria; Betaproteobacteriales; Neisseriaceae; Conchiformibius	5
Bacteria; Proteobacteria; Gammaproteobacteria; Betaproteobacteriales; Neisseriaceae; Neisseria	2
Bacteria; Proteobacteria; Gammaproteobacteria; Betaproteobacteriales; Nitrosomonadaceae; MND1	23
Bacteria; Proteobacteria; Gammaproteobacteria; Betaproteobacteriales; SC-I-84	3
Bacteria; Proteobacteria; Gammaproteobacteria; Cellvibrionales; Cellvibrionaceae; Cellvibrio	59
Bacteria; Proteobacteria; Gammaproteobacteria; Cellvibrionales; Cellvibrionaceae; Cellvibrio	16259
Bacteria; Proteobacteria; Gammaproteobacteria; Enterobacteriales; Enterobacteriaceae	172
Bacteria; Proteobacteria; Gammaproteobacteria; Nitrosococcales; Methylophagaceae; Methylophaga	40
Bacteria; Proteobacteria; Gammaproteobacteria; Oceanospirillales; Halomonadaceae; Halomonas	14
Bacteria; Proteobacteria; Gammaproteobacteria; Oceanospirillales; Halomonadaceae; Zymobacter; uncultured gamma proteobacterium	1

Bacteria; Proteobacteria; Gammaproteobacteria; Pasteurellales; Pasteurellaceae; Frederiksenia	24
Bacteria; Proteobacteria; Gammaproteobacteria; Pasteurellales; Pasteurellaceae; Haemophilus	3
Bacteria; Proteobacteria; Gammaproteobacteria; Pasteurellales; Pasteurellaceae; Pasteurella	77
Bacteria; Proteobacteria; Gammaproteobacteria; Pasteurellales; Pasteurellaceae	24
Bacteria; Proteobacteria; Gammaproteobacteria; Pseudomonadales; Moraxellaceae; Acinetobacter	593
Bacteria; Proteobacteria; Gammaproteobacteria; Pseudomonadales; Moraxellaceae; Enhydrobacter	3
Bacteria; Proteobacteria; Gammaproteobacteria; Pseudomonadales; Moraxellaceae; Moraxella	81
Bacteria; Proteobacteria; Gammaproteobacteria; Pseudomonadales; Pseudomonadaceae; Pseudomonas	398
Bacteria; Proteobacteria; Gammaproteobacteria; Xanthomonadales; Xanthomonadaceae; Luteimonas	5
Bacteria; Proteobacteria; Gammaproteobacteria; Xanthomonadales; Xanthomonadaceae; Stenotrophomonas	377
Bacteria; Proteobacteria; Gammaproteobacteria; Xanthomonadales; Xanthomonadaceae; Xanthomonas	11
Bacteria; Proteobacteria; Gammaproteobacteria; Xanthomonadales; Xanthomonadaceae	79
Bacteria; Spirochaetes; Spirochaetia; Spirochaetales; Spirochaetaceae; Sphaerochaeta; uncultured bacterium	19
Bacteria; Spirochaetes; Spirochaetia; Spirochaetales; Spirochaetaceae; Sphaerochaeta	23
Bacteria; Verrucomicrobia; Verrucomicrobiae; Opitutales; Puniceicoccaceae; Cerasicoccus; uncultured bacterium	40
Unassigned	3

Table S2: Percent of total feeding time each species was observed consuming each major food group, according to representative feeding behavior studies.

Species	Fruit	Leaves	Insect	Bamboo	Gum	Flower	Citation
Avahi peyrierasi	0	100	0	0	0	0	Faulkner & Lehman 2005
Cheirogaleus medius	60	0	38	0	0	2	Fietz & Ganzhorn 1999
Eulemur rubriventer	81	16	0	0	0	4	Overdorff 1993
Eulemur rufifrons (east)	70	26	0	0	0	4	Overdorff 1993
Eulemur rufifrons (west)	20	50	0	0	0	30	de Winter et al. 2013
Eulemur hybrids	89	10	0	0	0	1	Johnson 2002
Lemur catta	37	63	0	0	0	0	Simmen et al. 2003
Microcebus murinus	24	0	61	0	14	1	Dammhahn & Kappeler 2008
Microcebus rufus	44	0	54	0	2	0	Atsalis 1998
Mirza coquereli	40	0	60	0	0	0	Rowe et al. 2021
Phaner pallescens	4	0	8	0	86	2	Schülke 2003
Prolemur simus	2	3	0	95	0	0	Tan 1999
Propithecus edwardsi	21	76	0	0	0	3	Matos 2017
Propithecus verreauxi	0	80	0	0	0	20	de Winter et al. 2013
Varecia variegata	97	1.5	0	0	0	1.5	Donohue et al. 2019

Table S3: Co-dendrogram results. Column headers refer to datasets; R1-10 include random samples, "Aggregate" includes the mean ceiling dataset. Cell values are RF-distances*, which* range from 0 to 1, with 0 showing complete congruence between the GM dendrogram and host phylogeny and 1 showing complete incongruence.

**Metric**	**Dataset**										
	**R1**	**R2**	**R3**	**R4**	**R5**	**R6**	**R7**	**R8**	**R9**	**R10**	**Aggregate**
Bray-Curtis	0.67*	0.67*	0.67*	0.75*	0.58*	0.58*	0.58*	0.67*	0.67*	0.50*	0.67*
Jaccard	0.67*	0.67*	0.83*	0.67*	0.50*	0.50*	0.58*	0.67*	0.67*	0.50*	0.58*
Unweighted UniFrac	0.67*	0.50*	0.75*	0.67*	0.67*	0.58*	0.50*	0.67*	0.67*	0.67*	0.75*
Weighted UniFrac	0.92*	0.75*	0.92*	0.92*	0.92*	0.75*	0.75*	0.83*	0.67*	0.75*	0.75*

*p-value ≤ 0.05

Table S4: Mantel test results. Column headers refer to datasets; R1-10 include random samples, "Aggregate" includes the mean ceiling dataset. Cell values are R-values, which range from 0 to 1, with 0 showing no correlation between GM and host phylogenetic distance, and 1 showing complete congruence.

	**Dataset**										
**Metric**	**R1**	**R2**	**R3**	**R4**	**R5**	**R6**	**R7**	**R8**	**R9**	**R10**	**Aggregate**
Bray-Curtis	0.61**	0.51*	0.71**	0.17	0.60**	0.58*	0.51*	0.59**	0.50**	0.57*	0.49**
Jaccard	0.57**	0.53*	0.62**	0.22*	0.55**	0.51*	0.54*	0.60**	0.56**	0.69**	0.67**
Unweighted UniFrac	0.72**	0.42**	0.57**	0.72**	0.43*	0.70**	0.34*	0.37*	0.23*	0.76**	0.68**
Weighted UniFrac	0.55**	0.60**	0.56**	0.31*	0.65**	0.50*	0.47**	0.17	0.55**	0.55**	0.56**

*p-value ≤ 0.05
**p-value ≤ 0.001

Table S5: Measures of phylogenetic signal across diversity metrics. Column headers refer to datasets; R1-10 include random samples, "Aggregate" includes the mean ceiling dataset. For Blomberg's K, a value of one indicates the trait is evolving under Brownian Motion and exhibits strong phylogenetic signal; values closer to zero indicate no phylogenetic signal, and values above 1 indicate stronger trait similarity among closely related species than expected under Brownian Motion. Pagel's Lambda values cannot exceed one, which indicates the trait has strong phylogenetic signal and evolves under Brownian Motion. Values below one do not indicate phylogenetic signal.

Metric	Axis	Test	Dataset										
			**R1**	**R2**	**R3**	**R4**	**R5**	**R6**	**R7**	**R8**	**R9**	**R10**	**Aggregate**
Bray-Curtis	PCo1	Blomberg's K	1.89**	2.71**	1.65**	0.77*	2.45**	2.87**	1.88**	2.26**	2.64**	2.44**	2.52**
		Pagel's Lambda	0.94**	1.00**	1.00**	0.70**	1.00**	1.00**	0.95**	1.00**	1.00**	0.99**	1.00**
	PCo2	Blomberg's K	1.34**	1.94**	1.01*	0.20	1.95**	1.71**	1.88**	1.55**	1.79**	1.78**	1.96**
		Pagel's Lambda	1.00**	1.00**	0.98*	<0.10	1.00**	1.00**	1.00**	1.00**	1.00**	1.00**	1.00**
	PC3	Blomberg's K	0.77*	0.82*	0.84*	1.24**	0.28	1.00*	0.73*	1.04*	1.00*	0.94*	1.02*
		Pagel's Lambda	1.00	1.00	1.00	1.00*	<0.10	1.00*	1.00	1.00*	1.00*	1.00*	1.00*
	PC4	Blomberg's K	0.95*	0.81*	0.59	0.70	0.77*	0.69	0.90*	0.49	0.90*	0.46	0.62
		Pagel's Lambda	1.00*	1.00	<0.1	<0.10	1.00	<0.10	1.00*	<0.10	1.00*	<0.10	<0.10
	PC5	Blomberg's K	0.96*	0.90*	1.00*	0.89*	0.72*	0.65	0.49	0.93*	0.89*	0.86*	0.53
		Pagel's Lambda	1.00*	1.00*	1.00*	1.00*	<0.10	<0.10	<0.10	1.00*	1.00*	1.00	<0.10
Jaccard	PC1	Blomberg's K	1.49*	2.53**	1.68**	0.86*	1.88**	2.25**	2.06**	2.32**	2.60**	2.36**	2.30**
		Pagel's Lambda	0.89**	1.00**	1.00**	0.74*	0.92**	0.98**	0.97**	1.00**	1.00**	0.99**	0.98**
	PC2	Blomberg's K	1.29**	1.89**	1.05*	0.19	1.38*	1.76**	1.77**	1.70**	1.71**	1.62**	1.49**
		Pagel's Lambda	0.98*	1.00**	1.00*	<0.10	0.96**	1.00**	1.00**	1.00**	1.00**	1.00**	1.00**
	PC3	Blomberg's K	0.72*	0.99*	0.71	1.10*	0.30	0.95*	0.90*	1.30**	0.95*	0.84*	0.73*
		Pagel's Lambda	<0.10	1.00*	1.00	1.00*	<0.10	1.00*	1.00*	1.00*	1.00*	0.96	<0.10
	PC4	Blomberg's K	0.94*	0.72	0.87*	0.77	0.50	0.36	0.79*	0.97*	0.89*	0.51	0.33
		Pagel's Lambda	0.99*	<0.10	1.0	1.00	<0.10	<0.10	1.00	1.00*	1.00*	<0.10	<0.10
	PC5	Blomberg's K	0.65	1.09*	0.94*	0.91*	0.95*	1.01*	0.55	0.50	0.97*	1.00*	0.80*
		Pagel's Lambda	0.83	1.00*	1.00*	1.00*	1.00*	0.99*	<0.10	<0.10	1.00*	1.00*	1.00
Unweighted UniFrac	PC1	Blomberg's K	1.55**	2.08**	1.76**	0.94*	1.83**	2.16**	2.23**	2.03**	1.95**	1.52**	2.27**
		Pagel's Lambda	0.93**	1.00**	1.00**	0.83*	1.00**	1.00**	1.00**	1.00**	1.00**	1.00**	1.00**
	PC2	Blomberg's K	2.60**	2.53**	1.26*	1.87**	2.55**	3.03**	1.30*	2.49**	2.70**	1.83**	2.82**
		Pagel's Lambda	1.00**	1.00**	1.00*	0.95**	1.00**	1.00**	1.00*	1.00**	1.00**	0.97**	1.00**
	PC3	Blomberg's K	0.61	1.07*	0.65	0.48	0.62	0.73	0.79	1.11**	0.98*	0.81	0.65

		Pagel's Lambda	<0.10	1.00*	0.15	<0.10	<0.10	1.00	1.00	1.00*	1.00*	1.00	<0.10
	PC4	Blomberg's K	0.45	0.91*	1.00*	0.43	0.60	0.64	0.83*	0.92*	0.40	0.51	0.75*
		Pagel's Lambda	<0.10	1.00*	1.00*	0.44	<0.10	<0.10	1.00	1.00*	<0.10	<0.10	1.00
	PC5	Blomberg's K	0.60	0.84*	0.67	0.43	0.52	0.44	0.72	0.66	0.73	0.46	0.69
		Pagel's Lambda	<0.10	1.00	0.96	<0.10	<0.10	<0.10	1.00	<0.10	0.96	<0.10	<0.10
Weighted UniFrac	PC1	Blomberg's K	0.64	0.93*	0.39	0.65	0.99*	0.72*	0.68	1.53**	0.70	0.82*	1.35*
		Pagel's Lambda	0.93**	1.00*	0.35	0.56	0.90*	0.83	0.52	1.00**	<0.1	0.81*	1.00**
	PC2	Blomberg's K	0.95*	1.28**	0.68	0.40	0.78*	1.25**	0.78*	0.66	0.91*	0.74	0.88*
		Pagel's Lambda	1.00**	1.00*	0.60	0.20	0.83	1.00*	1.00	0.75	1.0*	1.0	1.00*
	PC3	Blomberg's K	0.95*	0.88*	0.82*	0.45	0.68	1.07*	0.68	0.90*	0.70	0.73	0.88*
		Pagel's Lambda	<0.10	1.00	1.00	<0.10	0.19	1.00*	0.51	1.00*	0.82	1.0	1.00*
	PC4	Blomberg's K	0.57	1.25**	0.77*	1.59**	0.85*	0.92*	1.33*	0.85*	1.44**	0.80*	1.05*
		Pagel's Lambda	<0.10	1.00*	0.97	1.00**	1.00	0.98*	0.97*	0.86*	1.0**	0.93	1.00*
	PC5	Blomberg's K	0.70	1.21*	0.78*	0.42	0.78*	0.76*	0.69	0.68	0.78*	1.24*	1.07*
		Pagel's Lambda	<0.10	1.00*	0.83	<0.10	1.00	1.00	0.69	0.91	1.0	1.0*	1.00*
Faith's PD	N/A	Blomberg's K	0.41	0.75*	0.51	0.68	1.00*	0.85*	0.97*	1.13**	0.39	0.66	1.21**
		Pagel's Lambda	0.21	0.86	0.36	0.63	1.00*	1.00	1.00*	0.91*	0.17	0.63	0.96*

*p-value ≤ 0.05
**p-value ≤ 0.001

Table S6: Adonis results show significant effects of host taxonomy, diet, and ecosystem on lemur GM beta diversity. Note diet and habitat are represented by PC1 scores.

**Beta diversity metric**	**Variable**	**Adonis Test**	
		**R^2**	**P-value**
Bray-Curtis	Family	0.18	0.001**
	Family:Genus	0.22	0.001**
	Family:Genus:Species	0.02	0.001**
	Diet	0.07	0.001**
	Habitat	0.05	0.001**
	Diet*Habitat	0.04	0.001**
Jaccard	Family	0.16	0.001**
	Family:Genus	0.20	0.001**
	Family:Genus:Species	0.02	0.001**
	Diet	0.06	0.001**
	Habitat	0.04	0.001**
	Diet*Habitat	0.04	0.001**
Unweighted UniFrac	Family	0.21	0.001**
	Family:Genus	0.16	0.001**
	Family:Genus:Species	0.01	0.001**
	Diet	0.13	0.001**
	Habitat	0.03	0.001**
	Diet*Habitat	0.02	0.001**
Weighted UniFrac	Family	0.18	0.001**
	Family:Genus	0.17	0.001**
	Family:Genus:Species	0.01	0.001**
	Diet	0.15	0.001**
	Habitat	0.02	0.001**
	Diet*Habitat	0.03	0.001**

**p-value $\leq$ 0.001

Table S7: Results of homogeneity of variance for Adonis results.

Beta diversity metric	Variable	Homogeneity of variance	
		**F-value**	**P-value**
Bray-Curtis	Family	2.06	0.146
	Genus	6.87	0.038*
	Species	5.97	0.787
	Diet	11.76	0.056
	Habitat	0.96	0.333
Jaccard	Family	0.79	0.531
	Genus	5.27	0.239
	Species	6.95	0.985
	Diet	12.05	0.125
	Habitat	0.92	0.305
Unweighted UniFrac	Family	11.24	0.001**
	Genus	14.16	0.001**
	Species	11.82	0.001**
	Diet	4.87	0.119
	Habitat	1.29	0.243
Weighted UniFrac	Family	3.74	0.025
	Genus	4.09	0.001**
	Species	4.171	0.003*
	Diet	3.27	0.043*
	Habitat	1.13	0.29

*p-value ≤ 0.05
**p-value ≤ 0.001

Table S8: Comparing the strength of trait correlations across four beta diversity metrics and one alpha diversity metric (Faith's phylogenetic distance). For these analyses, only the eigenvalues associated with the first principal components (i.e., PC1) for GM beta diversity (see "metric" column) and ecological traits (see "predictor p-value" columns) were considered. Overall, these results show diet is a significant predictor of GM beta diversity using UniFrac metrics.

Metric	Dataset	Predictor p-value			Model p-value	Model AIC value
		Diet	Habitat	Diet*Habitat		
Bray-Curtis	Host species aggregate	0.364	0.611	0.721	0.52	-9.17
	Random 1	0.444	0.191	0.148	0.07	-13.46
	Random 2	0.482	0.709	0.69	0.716	-10.89
	Random 3	0.773	0.732	0.534	0.740	-4.34
	Random 4	0.049*	0.249	0.839	0.028*	-3.45
	Random 5	0.178	0.559	0.636	0.254	-12.94
	Random 6	0.371	0.861	0.816	0.688	-10.94
	Random 7	0.285	0.446	0.72	0.315	-8.28
	Random 8	0.40	0.633	0.717	0.581	-10.08
	Random 9	0.548	0.871	0.791	0.862	-9.63
	Random 10	0.271	0.461	0.978	0.282	-13.73
Jaccard	Host species aggregate	0.503	0.316	0.84	0.37	-11.97
	Random 1	0.105	0.181	0.753	0.044*	-13.50
	Random 2	0.391	0.724	0.703	0.632	-12.13
	Random 3	0.613	0.469	0.548	0.433	-7.41
	Random 4	0.123	0.128	0.68	0.037*	-4.99
	Random 5	0.204	0.247	0.637	0.23	-12.76
	Random 6	0.212	0.527	0.888	0.271	-13.80
	Random 7	0.423	0.381	0.615	0.395	-9.45
	Random 8	0.496	0.553	0.743	0.617	-10.73
	Random 9	0.613	0.735	0.88	0843	-10.91
	Random 10	0.287	0.443	0.933	0.279	-14.78
Unweighted UniFrac	Host species aggregate	0.021*	0.419	0.574	0.09	-21.05
	Random 1	0.001**	0.99	0.94	0.003*	-26.54
	Random 2	0.06	0.11	0.51	0.23	-17.81
	Random 3	0.59	0.97	0.58	0.81	-12.15

	Random 4	0.002*	0.74	0.41	0.006*	-15.41
	Random 5	0.007*	0.02*	0.56	0.04*	-21.86
	Random 6	0.015*	0.28	0.97	0.09	-20.57
	Random 7	0.016*	0.638	0.15	0.04*	-22.59
	Random 8	0.09	0.29	0.81	0.04*	-15.79
	Random 9	0.017*	0.08	0.84	0.09	-20.27
	Random 10	0.02*	0.26	0.47	0.12	-14.32
Weighted UniFrac	Host species aggregate	0.012*	0.488	0.747	0.01*	-33.69
	Random 1	0.052	0.047*	0.63	0.006*	-20.44
	Random 2	0.044*	0.307	0.173	0.099	-19.66
	Random 3	0.038*	0.006*	0.917	0.0006**	-17.95
	Random 4	0.028*	0.199	0.654	0.009*	-16.44
	Random 5	0.006*	0.884	0.31	0.011*	-30.17
	Random 6	0.169	0.147	0.29	0.0616	-17.51
	Random 7	0.552	0.421	0.861	0.466	-10.27
	Random 8	0.52	0.679	0.654	0.890	-17.73
	Random 9	0.203	0.468	0.133	0.162	-10.49
	Random 10	0.093	0.828	0.469	0.134	-16.07
Faith's PD	Host species aggregate	0.005*	0.506	0.446	0.02*	73.92
	Random 1	0.003*	0.549	0.622	0.01*	97.52
	Random 2	0.04*	0.055	0.036*	0.14	98.38
	Random 3	0.853	0.02*	0.276	0.046*	99.13
	Random 4	0.025*	0.164	0.368	0.012*	87.54
	Random 5	0.029*	0.094	0.919	0.139	87.12
	Random 6	0.051	0.45	0.021*	0.021*	84.15
	Random 7	0.236	0.175	0.129	0.085	88.09
	Random 8	0.031*	0.64	0.471	0.116	80.99
	Random 9	0.130	0.385	0.571	0.393	102.94
	Random 10	0.002*	0.44	0.554	0.008*	90.82

*p-value ≤ 0.05
**p-value ≤ 0.001

Table S9: Pairwise comparisons of niche overlap calculated using the D metric, which quantifies similarity between species. The statistic ranges from 0 (no niche overlap) to 1 (identical niches; see Warren et al. 2008). Darker shading indicates sampling occurred in contrasting environments (i.e., one species was sampled in the drier west, while the other was sampled in the wetter east). Lighter shading indicates sampling occurred in similar environments (e.g., both species were sampled in the west).

SPECIES	CM	ERE	ERB	ERW	HY	LC	MM	MR	MC	PE	PP	PS	PV	VV	AP
***C. medius* (CM)**	1	-	-	-	-	-	-	-	-	-	-	-	-	-	-
***E. rufifrons* east (ERE)**	0.18	1	-	-	-	-	-	-	-	-	-	-	-	-	-
***E. rubriventer* (ERB)**	0.15	0.62	1	-	-	-	-	-	-	-	-	-	-	-	-
***E. rufifrons* west (ERW)**	0.27	0.06	0.04	1	-	-	-	-	-	-	-	-	-	-	-
**Hybrid (HY)**	0.37	0.61	0.51	0.13	1	-	-	-	-	-	-	-	-	-	-
***Lemur catta* (LC)**	0.27	0.17	0.14	0.32	0.33	1	-	-	-	-	-	-	-	-	-
***Mic. murinus* (MM)**	0.78	0.13	0.1	0.34	0.31	0.31	1	-	-	-	-	-	-	-	-
***Mic. rufus* (MR)**	0.12	0.76	0.56	0.03	0.49	0.13	0.08	1	-	-	-	-	-	-	-
***Mir. coquereli* (MC)**	0.71	0.1	0.04	0.33	0.2	0.24	0.68	0.04	1	-	-	-	-	-	-
***Prop. edwardsi* (PE)**	0.04	0.44	0.36	0	0.23	0.04	0.02	0.56	0	1	-	-	-	-	-
***Ph. pallescens* (PP)**	0.25	0.02	0	0.42	0.07	0.03	0.31	0.01	0.32	0	1	-	-	-	-
***Prol. simus* (PS)**	0.1	0.66	0.6	0.01	0.42	0.06	0.07	0.68	0.01	0.46	0	1	-	-	-
***Prop. verreaui* (PV)**	0.45	0.08	0.05	0.46	0.22	0.33	0.54	0.05	0.42	0.01	0.27	0.02	1	-	-
***V. variegata* (VV)**	0.17	0.55	0.64	0.03	0.46	0.06	0.13	0.52	0.05	0.3	0.02	0.71	0.05	1	-
***A. peyrierasi* (AP)**	0.25	0.72	0.64	0.07	0.71	0.17	0.19	0.56	0.12	0.28	0.03	0.59	0.11	0.65	1

Table S10: Area under curve (AUC) values for each species' habitat suitability model. AUC > 0.8 is considered informative.

Species	AUC value	Standard deviation
Avahi peyrierasi	0.951	0.046
Cheirogaleus medius	0.823	0.097
Eulemur rubriventer	0.937	0.040
Eulemur rufifrons (east)	0.956	0.060
Eulemur rufifrons (west)	0.956	0.038
Eulemur hybrids	0.946	0.026
Lemur catta	0.975	0.013
Microcebus murinus	0.946	0.872
Microcebus rufus	0.964	0.031
Mirza coquereli	0.849	0.132
Phaner pallescens	0.991	0.002
Propithecus edwardsi	0.996	0.001
Propithecus verreaui	0.950	0.028
Varecia variegata	0.955	0.049

Supporting Information Works Cited

Atsalis S. 1999. Diet of the brown mouse lemur (*Microcebus rufus*) in Ranomafana National Park, Madagascar. *International Journal of Primatology.* 20:193-229. DOI:10.1023/A:1020518419038

Dammhahn M & Kappeler PM. 2008. Comparative feeding ecology of sympatric *Microcebus berthae* and *M. murinus. International Journal of Primatology.* 29:1567-1589. DOI:10.1007/s10764-008-9312-3

de Winter II, Gollner A, Akom E. 2013. Diet overlap of *Propithecus verreauxi* and *Eulemur rufifrons* during the late dry season in Kirindy Forest. *Lemur News.* 17:18-21.

Donohue ME, Absanga AE, Ralainirina J, Weisrock DW, Stumpf RM, Wright PC. 2019. Extensive variability in the gut microbiome of a highly-specialized and critically endangered lemur species across sites. *American Journal of Primatology.* DOI:10.1002/ajp.23046

Faulkner AL & Lehman SM. 2006. Feeding patterns in a small-bodied nocturnal folivore (*Avahi laniger*) and the influence of leaf chemistry: a preliminary study. *Folia Primatologica.* 77:218-227. DOI:10.1159/000091231

Fietz J & Ganzhorn JU. 1999. Feeding ecology of the hibernating primate *Cheirogaleus medius*: how does it get so fat? *Oecologia.* 121:157-164. DOI:10.1007/s004420050917

Hijmans RJ, Cameron SE, Parra JL, Jones PG, Jarvis A. 2005. Very high resolution interpolated climate surfaces for global land areas. *International Journal of Climatology.* 25:1965-1978. DOI:10.1002/joc.1276

Hosmer DW & Lemeshow S. 2000. *Applied Logistic Regression,* 2nd edition. John Wiley & Sons, New York.

Johnson SE. 2002. Ecology and speciation in brown lemurs: white-collared lemurs (*Eulemur albocollaris*) and hybrids (*Eulemur albocollaris* x *Eulemur fulvus rufus*) in southeastern Madagascar. PhD Dissertation, The University of Texas at Austin.

Matos MDP. 2017. Comparing the feeding ecology of *Propithecus edwardsi* in disturbed and undisturbed forest in Ranomafana National Park, Madagascar. Masters Thesis, Universidade de Évora.

Overdorff DJ. 1993. Similarities, differences, and seasonal patterns in the diets of *Eulemur rubriventer* and *Eulemur fulvus rufus* in the Ranomafana National Park, Madagascar. *International Journal of Primatology.* 14(5):721-753. DOI:10.1007/BF02192188

Phillips SJ, Anderson RP, Dudík M, Schapire RE, Blair ME. 2017. Opening the black box: an open-source release of Maxent. *Ecography.* 40:887-893. DOI:10.1111/ecog.03049

Rowe AK, Donohue ME, Clare EL, Drinkwater R, Koenig A, Ridgway ZM, Martin LD, Nomenjanahary ES, Zakamanana F, Randriamanandaza LJ, Rakotonirina TE, Wright PC. Exploratory analysis reveals arthropod consumption in ten lemur species using DNA metabarcoding. *American Journal of Primatology. In press.*

Schülke O. 2003. To breed or not to breed – food competition and other factors involved in female breeding decisions in the pair-living nocturnal fork-marked lemur (*Phaner furcifer*). *Behavioral Ecology and Sociobiology.* 55:11-21. DOI:10.1007/s00265-003-0676-2

Simmen B, Hladik A, Ramasiarisoa P. 2003. Food intake and dietary overlap in native *Lemur catta* and *Propithecus verreauxi* and introduced *Eulemur fulvus* at Berenty, Southern Madagascar. *International Journal of Primatology.* 5(2):949-968.

Tan CL. 1999. Group composition, home range size, and diet of three sympatric bamboo lemur species (Genus *Hapalemur*) in Ranomafana National Park, Madagascar. *International Journal of Primatology.* 20:547-566. DOI:10.1023/A:1020390723639

Warren DL, Glor RE, Turelli M. 2008. Environmental niche equivalency versus conservatism: quantitative approaches to niche evolution. *Evolution.* 62(11):2868-2883. DOI:10.1111/j.1558-5646.2008.004832.x
